# Two cancer cell lines utilize Myosin 10 and the kinesin HSET differentially to maintain mitotic spindle bipolarity

**DOI:** 10.1371/journal.pone.0325016

**Published:** 2025-05-29

**Authors:** Yang-In Yim, Xufeng Wu, Anjelika Gasilina, John A. Hammer

**Affiliations:** Cell and Developmental Biology Center, National Heart Lung and Blood Institute, National Institutes of Health, Bethesda, Maryland, United States of America; Centre de Recherche en Biologie cellulaire de Montpellier, FRANCE

## Abstract

Cancer cells often undergo mitosis possessing more than two centrosomes. To avoid a multipolar mitosis, the consequences of which are typically aneuploidy induced senescence, they must cluster their extra centrosomes to create a pseudo-bipolar spindle. Such supernumerary centrosome clustering (SNCC) requires Myosin 10 (Myo10) and the pole-focusing kinesin HSET. We showed recently that Myo10 promotes SNCC in HeLa cells by promoting retraction fiber-based cell adhesion, and that it further supports spindle bipolarity by preventing the generation of extra spindle poles via pericentriolar material (PCM) fragmentation. Here we quantified the contribution that Myo10 and HSET make individually and together to SNCC and PCM/pole integrity in HeLa cells and in MDA-MB-231 cells, which differ from HeLa in being more dependent on SNCC and less dependent on retraction fiber-based cell adhesion. As expected, knockdown of Myo10 and HSET individually increased the frequency of multipolar spindles in both cell types. Their effects were surprisingly not additive, however. For HeLa and MDA-MB-231 cells undergoing mitosis with more than two centrosomes, the defect in SNCC was almost entirely responsible for their multipolar phenotype following knockdown of either Myo10 or HSET. For HeLa and MDA-MB-231 cells undergoing mitosis with two centrosomes, PCM/pole fragmentation was the primary cause of multipolar spindles following HSET knockdown. Unlike HeLa, however, MDA-MB-231 cells exhibited very little PCM/pole fragmentation following Myo10 knockdown. This difference may be due to the smaller role that Myo10 plays in retraction fiber-based adhesion in MDA-MB-231. Finally, we show that HSET knockdown disrupts retraction fiber formation and organization, which may explain why the defects in double knockdown cells were not significantly greater than in HSET knockdown cells. These and other results can inform efforts to target these two motor proteins to selectively kill cancer cells by increasing their frequency of multipolar divisions.

## Introduction

The bipolar nature of the mitotic spindle plays an essential role in the accurate partitioning of chromosomes into daughter cells [1–5]. The two poles of the bipolar spindle are created during the S and G2 phases of the cell cycle by duplication of a single G1 centrosome. If cells possess more than one G1 centrosome, the duplication process will result, therefore, in more than two spindle poles. Failure to address this issue prior to anaphase will usually result in a multipolar mitosis, the common consequence of which is inaccurate segregation of chromosomes to daughter cells (i.e., aneuploidy) followed by their senescence [6–10]. Supernumerary centrosomes can originate from centrosome overduplication, de novo synthesis of centrosomes, cytokinesis failure, or cell: cell fusion [11]. Extra mitotic spindle poles can also be created in the absence of centrosome amplification through disengagement of one of the two centrioles within a pole or by fragmentation of the pericentriolar material (PCM) that encases the centrioles, as both can create aberrant microtubule organizing centers (MTOCs) that capture centromeres [11]. While these issues are very uncommon in normal cells, they are quite common in many cancer cell types [6–10]. To survive, therefore, such cancer cells must be capable of clustering their extra spindle poles into two groups to enable a pseudo-bipolar mitosis. Such clustering is commonly referred to as a supernumerary centrosome clustering (SNCC). Given that supernumerary centrosomes are rare in normal cells and common in both solid tumor and hematological cancer cells, drugs that inhibit SNCC should selectively kill cancer cells [6–10].

A genome-wide siRNA-based screen in near tetraploid Drosophila S2 cells for proteins that suppress multipolar mitoses identified two motor proteins that promote SNCC [12] (in addition to dynein which was shown previously to be required for SNCC [13]). One, the MT minus end-directed kinesin 14 family member NCD, fit nicely with the fact that it was already known to drive the focusing of meiotic spindle poles in fly oocytes [14]. The other, Myosin 10a, is the fly homolog of the mammalian MyTH4/FERM myosin family member Myosin 15. This result was interesting given that Myosin 10 (Myo10), another mammalian MyTH4/FERM myosin family, is in principle capable of coupling microtubules and integrin-based adhesions to movement along actin filaments via its microtubule-binding MyTH4 domain and its integrin-binding FERM domain, respectively [15–19].

Consistent with the results of the S2 cell screen, RNAi-mediated knockdown (KD) of HSET, the mammalian homolog of NCD, in MDA-MB-231 breast cancer cells (which exhibit supernumerary centrosomes about 35% of the time) inhibited SNCC [12]. This result is also consistent with recent evidence that HSET (also known as KIFC1) drives the focusing of acentrosomal meiotic spindle poles in mammalian eggs [20]. Also consistent with the Drosophila screen, RNAi-mediated KD of Myo10 in MDA-MB-231 cells inhibited SNCC [12]. Notably, these effects were unique to MDA-MB-231 cells with extra centrosomes/poles, as both KDs did not induce multipolar spindles in cells undergoing mitosis with two centrosomes. In terms of the mechanism by which Myo10 supports SNCC, a subsequent study from this same lab showed that Myo10 harboring a mutated MyTH4 domain that can no longer bind to microtubules could not rescue the multipolar phenotype exhibited by Myo10 KD HeLa cells that possess extra spindle poles due to the over overexpression of PLK4 [21].

While Kwon et al did not perform direct tests for synergy between HSET and Myo10 by depleting both motors simultaneously, at least some degree of synergy seems likely given that assays quantifying the suppression of multipolar spindles showed that actin disassembly was not synergistic with Myo10 KD (presumably because they act in the same pathway), but was synergistic with HSET KD [12]. Indeed, synergy would be expected because these two motor proteins likely support distinct aspects of tension required for SNCC, with Myo10 supporting actin-based adhesion tension and HSET supporting microtubule-based spindle tension [7]. Finally, it is important to note in the context of the present study that the S2 cell screen also identified several proteins involved in cell: ECM adhesion as suppressors of multipolar mitoses. Consistently, Kwon et al showed using micropatterning of ECM components that the ability of MDA-MB-231 cells to cluster their supernumerary centrosomes is influenced significantly by the pattern of adhesion [12].

We recently used genome-edited HeLa cells that are almost devoid of Myo10 to define the contribution that this motor protein makes to maintaining spindle bipolarity [22]. We also used complementation of these cells with mutated versions of Myo10 to quantitate the contributions that its microtubule-binding MyTH4 and integrin-binding FERM domains make to the myosin’s ability to maintain spindle bipolarity. Finally, we used HeLa cells in which one Myo10 allele was endogenously tagged with Halo using CRISPR to obtain definitive information on the myosin’s localization during mitosis. We found that Myo10 depleted HeLa cells exhibit a pronounced increase in the frequency of multipolar spindles, and that two separate defects are responsible. The first defect, spindle pole fragmentation, is the major driver of multipolar spindles in Myo10 depleted HeLa cells lacking supernumerary centrosomes. We showed that pole fragmentation is due almost entirely to PCM fragmentation (centriole disengagement plays a minor role), that fragmentation occurs as cells approach metaphase (arguing that it is force-dependent), and that the recruitment of two pole maturation markers is normal. While we did not see Myo10 at poles, we did see a faint signal in the spindle that depends on the myosin’s ability to bind to microtubules. Finally, complementation experiments showed that Myo10 must interact with both integrins and microtubules to promote pole stability. Together, these results indicated that the defect in PCM integrity is not a direct result of losing Myo10 at spindle poles, nor is it due to a general defect in spindle pole maturation. Instead, our results suggested that Myo10 promotes PCM/pole integrity through a MyTH4 domain-dependent interaction with spindle microtubules and a FERM-dependent interaction with integrins [22].

The second defect, an inability to cluster extra spindle poles, is the major driver of multipolar spindles in Myo10 depleted HeLa cells possessing supernumerary centrosomes [22]. Unlike the defect in PCM/pole stability, Myo10 only needs interact with integrins to promote SNCC. Consistently, endogenously tagged Myo10 localizes dramatically to the tips of retraction fibers, which are known to support integrin-based adhesion during mitosis [23–29]. These and other results argued that Myo10’s FERM domain-dependent interaction with ECM-bound integrins at the tips of retraction fibers promotes SNCC by serving as an anchor for the microtubule-based pole focusing forces that drive clustering [22].

Here we used siRNA-mediated KD of Myo10 and HSET individually and together to quantify their sole and combined contributions to SNCC and PCM/pole integrity in HeLa and MDA-MB-231 cells. We chose to include both cell lines in our study because previous efforts to define mechanisms of SNCC in HeLa [21,22] and MDA-MB-231 [12] did not provide direct comparisons. We reasoned that directly comparing these two cancer cell lines could have value given that they differ significantly in their dependence on SNCC (~35% of MDA-MB-231 cells possess supernumerary centrosomes versus ~15% of HeLa cells [12,22,30]). Direct comparisons could also have value given that they differ significantly in their migratory phenotype (MDA-MB-231 cells are significantly more migratory than HeLa cells [31]). Perhaps related to this later difference, we show here that MDA-MB-231 cells are less dependent than HeLa cells on RF-based adhesion during mitosis, which as noted above is the primary pathway by which Myo10 supports SNCC in HeLa cells. Our results show that HeLa and MDA-MB-231 cells utilize Myo10 and HSET differentially to avoid multipolar mitoses. These and other results can inform efforts to target Myo10 and HSET to selectively kill cancer cells by increasing their frequency of multipolar divisions [6–10].

## Materials and methods

### Cell culture, siRNA-mediated KD, and chemicals

HeLa cells (ATCC, CCL-2), MDA-MB-231 cells (ATCC, HTB-26), and Myo10 KO-1 cells [22] were cultured in high glucose DMEM (Gibco) supplemented with 10% FBS (Gibco) and Antibiotic-Antimycotic solution (Gibco) at 37°C in a 5% CO_2_. For imaging purposes, cells were cultured in coverglass bottom chamber slides (Cellvis) coated with fibronectin (10 μg/ml; Gibco). To knockdown Myo10 and/or HSET, WT HeLa and MDA-MB-231 cells were transfected with ON-TARGETplus human Myo10 siRNA-SMARTpool (Dharmacon; L-007217–00) and/or ON-TARGETplus human KIFC1 siRNA-SMARTpool (Dharmacon; L-004958–00) using Lipofectamine RNAiMAX reagent (Invitrogen). To increase Myo10 KD efficiency, transfection was performed twice in two day intervals and in reverse fashion. Only a single transfection was done for HSET, however, as the near-complete KD achieved using two transfections resulted in cell cycle arrest and significant cell death. All cells were checked routinely by PCR for mycoplasma contamination. The anti-HSET drug CW069 [32] was purchased from Selleckchem (57336) and treated at 100 μM concentration for 2hr.

### Antibodies and immunofluorescence

The following primary antibodies were used: mouse anti-γ-tubulin (MilliporeSigma, T6793, 1:300), mouse anti-α-tubulin (Abcam, ab7291, 1:300), rabbit anti-α-tubulin (Abcam, ab52866, 1:300), rabbit anti-myo10 (MilliporeSigma, HPA024223, 1:300), mouse anti-centrin1 (MilliporeSigma, 04−1624, 1:200), rabbit anti-centrin-1 (Abcam, ab101332, 1:200), rabbit anti-HSET (Abcam, ab172620, 1:200), and rabbit anti-Kizuna (ProteinTech, 21177–1-PP, 1:200). AlexaFluor-conjugated and HRP-conjugated secondary antibodies were purchased from Jackson ImmunoResearch Laboratories. DAPI and Alexa Fluor 488 labeled Phalloidin were purchased from ThermoFisher Scientific. Cells were fixed in 4% PFA for 10 min at RT unless subsequent staining was for centrosomes and pole-related proteins, in which case the cells were subjected to a two-step fixation method involving 1.5% PFA for 5 min followed by ice-cold MeOH for 5 min at −20°C. All PFA solutions were prepared in Cytoskeleton Stabilization Buffer (150 mM NaCl, 5 mM EGTA, 5 mM glucose, 5 mM MgCl_2_, and 10 mM PIPES (pH 6.8)). Fixed cells were permeabilized and blocked by incubation for 15 min in PBS containing 0.15% Saponin and 5% Goat serum. Fixed, permeabilized cells were incubated at RT for 1 hr with primary and secondary antibodies diluted in blocking solution, with an intervening wash cycle using PBS.

### Immunoblotting

Whole cell protein lysates were collected directly from culture dishes by adding 1X SDS sample buffer, as described previously [33]. Samples were resolved on 4–12% NuPAGE Bis-Tris gels (ThermoFisher Scientific) and transferred onto nitrocellulose membranes (Bio-Rad) using a semi-dry transfer system (Bio-Rad). Nitrocellulose membranes were blocked in TBST (10 mM Tris, pH 8.0, 150 mM NaCl, and 0.02% Tween 20) supplemented with 5% milk for 2 hrs, incubated with anti-Myo10 primary antibody (1:1000), anti-HSET primary antibody (1:1000), or anti-Kizuna primary antibody (1:1000) overnight at 4°C, washed with TBST, and incubated with HRP-conjugated secondary antibody (1:10,000) at RT for 2 hrs. Antibodies were diluted in TBST containing 5% milk. Actin detected using the anti-β-actin antibody (Abcam, ab6276, 1:10,000) was used as a loading control. Proteins were detected using SuperSignal West Pico Plus Chemiluminescent Substrate (ThermoFisher Scientific, 34577) and quantitated using an Amersham Imager 600 (GE Healthcare Life Sciences).

### Scoring cell phenotypes

To score mitotic phenotypes, HeLa and MDA-MB-231 cells were plated in imaging chambers at moderate density, cultured overnight, fixed, and stained with anti-γ-tubulin, anti-α-tubulin, anti-centrin-1, and DAPI. Of note, cells were never synchronized for these studies. Z-stack images were taken in 0.25 µm steps. The criteria used to score metaphase cells as bipolar, semi-bipolar, or multipolar are described in the text. To distinguish mitotic cells containing two centrosomes from mitotic cells containing supernumerary centrosomes, and to distinguish γ-tubulin-positive poles containing two centrioles from those containing one centriole or no centriole, metaphase cells were stained with anti-γ-tubulin, anti-centrin-1 and DAPI. Retraction fiber length was measured by tracing each RF using Image J. Retraction fiber straightness was determined by comparing linear end-to-end length versus contour path length as described previously [34]. The content of stress fibers under the nucleus was determined by outlining the DAPI-stained nucleus and then measuring the total intensity of the Fluor 488 labeled Phalloidin signal within this ROI using Image J. The distance between peripheral actin stress fibers and the plasma membrane was measured by drawing lines perpendicular to both using Image J. Each number was the mean of two longest lines, and measurements were restricted to regions that presented as leading edges (i.e., contained lamellipodial extensions). To measure the relative intensity of Kizuna staining at metaphase spindle poles, cells were fixed and stained for α-tubulin and Kizuna and imaged in 0.25 µm steps. The total intensity of Kizuna per pole in cells with bipolar spindles was determined by summing slices and then thresholding using the OTSU function in Image J.

### Imaging and statistical analyses

Imaging was performed on a Zeiss Airyscan 880 microscope equipped with a 60X, 1.4 NA objective. Images were processed in auto strength mode using ZenBlack software (Version 2.3) and analyzed using ImageJ. Excel or GraphPad Prism were used for statistical analyses and graphing. Statistical significance was determined using unpaired t-test and indicated as follows: * = P < 0.05, ** = P < 0.01, *** = P < 0.001, and **** = P < 0.0001.

## Results

### The localizations of Myo10 and HSET in both HeLa and MDA-MB-231 cells are consistent with Myo10 supporting adhesion-based tension and HSET supporting microtubule-based spindle tension during mitosis

**Fig 1:**
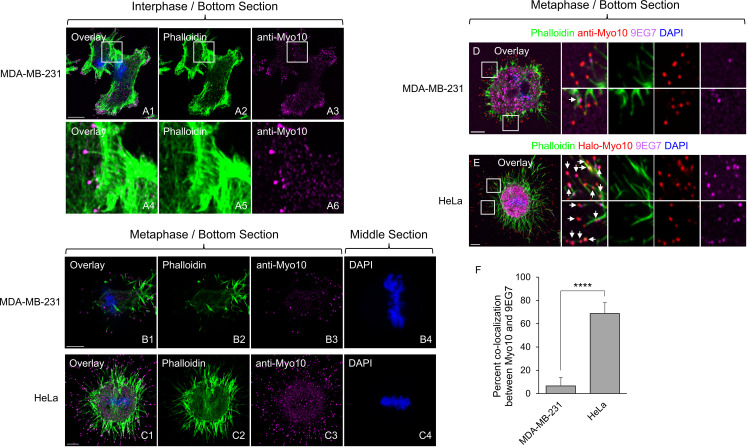
While Myo10 also localizes to the tips of mitotic retraction fibers in MDA-MB-231 cells, these structures are less organized and exhibit less colocalization between Myo10 and active **β1 integrin than retraction fibers in HeLa cells.** (A1-A6) Representative bottom section images of interphase MDA-MB-231 cells stained for actin (Phalloidin) and Myo10. (B1-B4) Representative images (B1-B3: bottom sections; B4: middle section) of metaphase MDA-MB-231 cells stained for actin (Phalloidin), Myo10 and DNA (DAPI). (C1-C4) Representative images (C1-C3: bottom sections; C4: middle section) of metaphase HeLa cells stained for actin (Phalloidin), Myo10 and DNA (DAPI). (D) Representative images of a metaphase MDA-MB-231 cell stained for actin (Phalloidin), Myo10, open, active β1 integrin (9EG7), and DNA (DAPI) showing the degree to which Myo10 puncta at the tips of retraction fibers exhibit a signal for open, active β1 integrin (the arrows in the insets mark Myo10-positive retraction fiber tips that also exhibit the integrin signal). (E) Same as (D) except a representative metaphase HeLa cell. (F) Percent co-localization between the signals for Myo10 and open, active integrin (9EG7) at the tips of retraction fibers (from 15 cells each). The mag bar in A1 is 10 µm, and the mag bars in B1, C1, D and E are 5 µm.

We previously used immunostaining with a highly-specific Myo10 antibody, low level over-expression of mScarlet tagged Myo10, and endogenous tagging of Myo10 with Halo using CRISPR-based knockin (KI) to localize Myo10 in HeLa cells [22]. All three methods showed that Myo10 localizes diffusely throughout the cytoplasm and robustly at the tips of ventral and dorsal filopodia during interphase. All three methods also showed that Myo10 localizes diffusely throughout the cytoplasm and robustly at the tips of dorsal filopodia and substrate-bound retraction fibers during metaphase. Moreover, this latter Myo10 signal was seen to overlap significantly with open, active integrin. Given this, given the extensive evidence that retraction fibers are essential for the integrin-dependent adhesion of mitotic cells [23–26,28,29,27], and given our complementation data showing that Myo10 only needs interact with integrins to promote SNCC [22], we concluded that Myo10’s ability to promote retraction fiber-based cell adhesion tension during mitosis via its FERM domain-dependent interaction with integrins plays the central role in its ability to promote SNCC [22]. Consistent with this mechanism, staining of MDA-MB-231 cells with the Myo10-specifc antibody shows that, as in HeLa cells, the myosin localizes robustly at the tips of filopodia during interphase (Fig 1 A1-A6) and at the tips of metaphase retraction fibers during mitosis (Fig 1 B1-B4) (although see below).

**Fig 2:**
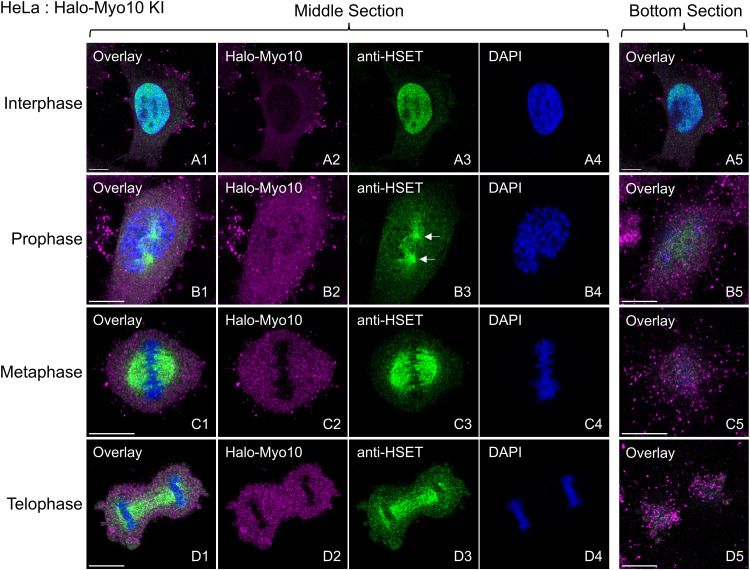
Endogenous HSET localizes in HeLa cells to the nucleus during interphase, to spindle poles during prophase, and to the spindle from metaphase through telophase. Representative images of Halo-Myo10 KI HeLa cells stained for HSET and DNA (DAPI) at interphase (A1-A5), prophase (B1-B5), metaphase (C1-C5), and telophase (D1-D5). Shown are middle sections and bottom sections. The white arrows in B3 mark the positions of the two spindle poles. All mag bars are 10 µm.

To localize HSET in HeLa, we stained our Halo-Myo10 KI HeLa cells with an anti-HSET antibody (see below for evidence that this antibody is HSET-specific). As in our previous study [22], endogenously tagged Myo10 localizes at the tips of dorsal and ventral filopodia during interphase (Fig 2A1, 2A2, 2A5; the Myo10-positive tips of ventral filopodia are clearest in the bottom section in A5) and at the tips of dorsal filopodia and ventral retraction fibers during mitosis (Fig 2B1, 2B2, 2B5, 2C1, 2C2, 2C5, 2D1, 2D2, 2D5; the Myo10-positive tips of retraction fibers are clearest in the bottom sections in B5, C5 and D5). Consistent with previous studies [35–37], HSET localizes within nuclei during interphase (Fig 2A1, 2A3–2A5), robustly to forming spindle poles during prophase (Fig 2B1, 2B3–2B5; see the white arrows in B3), and robustly to the spindle from metaphase (Fig 2C1, 2C3–2C5) through telophase (Fig 2D1, 2D3–2D5). Essentially identical results were obtained for interphase and mitotic MDA-MB-231 stained for HSET (S1 Fig). These localization data, together with previous work showing that this kinesin drives the focusing of acentrosomal meiotic spindle poles in fly [14] and mammalian [20] eggs, is consistent with the idea this microtubule minus end-directed kinesin drives SNCC at least in part by maintaining microtubule-based spindle tension [7].

### MDA-MB-231 cells do differ from HeLa cells with regard to retraction fiber morphology and the degree to which Myo10 and active integrin colocalize

While MDA-MB-231 cells are similar to HeLa cells with regard to the localization of Myo10 at the tips of metaphase retraction fibers, they differ significantly from HeLa with regard to retraction fiber morphology, with MDA-MB-231 cell retraction fibers being shorter, sparser and less straight than HeLa cell retraction fibers (Fig 1; compare B1-B4 to C1-C4; these images are representative of 88% of MDA-MB-31 cells (N = 76) and 90% of HeLa cells (N = 212)). A second significant difference between these two cell types has to do with the degree to which Myo10 puncta at the tips of retraction fibers exhibit a signal for open, active β1 integrin (detected by staining with antibody 9EG7), which is significantly lower in MDA-MB-231 cells than in HeLa cells (Fig 1; compare D to E; the arrows in the insets mark Myo10-positive retraction fiber tips that also exhibit a signal for open, active β1 integrin; for these two cells, the percent of Myo10 puncta that exhibited a signal for open, active β1 integrin was 7.2% for the MDA-MB-231 cell and 71.7% for the HeLa cell; the cumulative data from 15 cells was 6.6 ± 7.3% for MDA-MB-231 cells and 68.8 ± 9.4% for HeLa cells, Fig. 1F). Together, these results argue that Myo10’s contribution to adhesion-based tension during mitosis is likely significantly smaller in MDA-MB-231 cells than in HeLa cells. This difference, and the difference in the percent of cells that undergo mitosis with more than two centrosomes (~35% for MDA-MB-231 versus ~15% for HeLa; see below and [22,30], are particularly relevant for this study.

### Depletion of Myo10 and HSET individually increases the frequency of multipolar spindles in both HeLa and MDA-MB-231 cells, but their effects are surprisingly not additive

**Fig 3:**
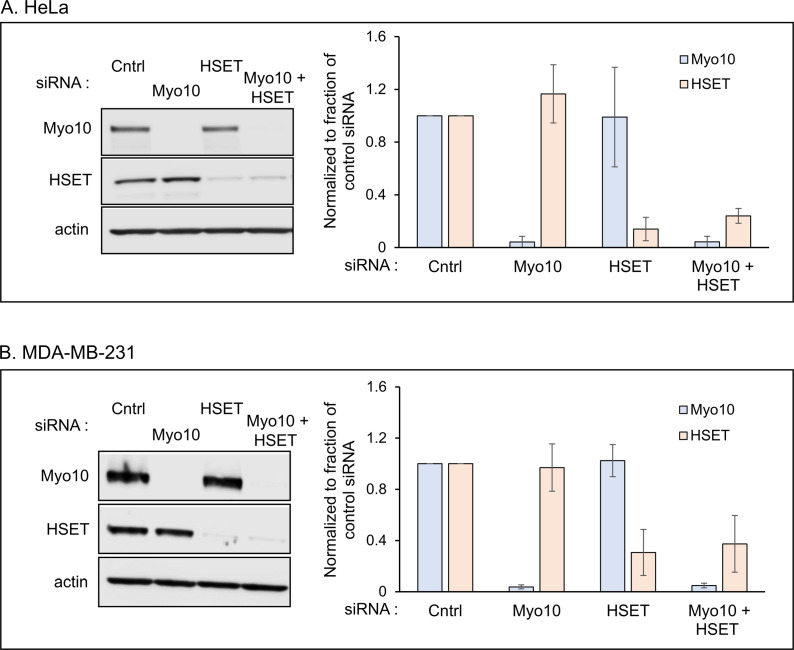
siRNA-mediated knockdown efficiencies for Myo10 and HSET individually and together in HeLa and MDA-MD-231 cells. (A) Representative Western blot and knockdown efficiencies for Myo10 (Light Blue) and HSET (Peach) in HeLa cells from four independent experiments (see also S1 Table). (B) Same as (A) but for MDA-MB-231 cells and from three independent experiments. See also S1 Table.

We used siRNA-mediated transient depletion of Myo10 and HSET to assess their roles individually and together in maintaining spindle bipolarity in HeLa and MDA-MB-231 cells (of note, Myo10 expression in MDA-MB-231 is 87.8 ± 3.7% that in HeLa, while HSET expression in MDA-MB-231 is 3.0 ± 0.2 fold higher than in HeLa; S2 Fig). Briefly, both cell types were transfected with either control non-targeting siRNA, ON-TARGETplus human Myo10 SMARTpool siRNA, ON-TARGETplus human HSET SMARTpool siRNA, or both Myo10 and HSET SMARTpool siRNAs (see Materials and Methods for details). To increase Myo10 knockdown (KD) efficiency, transfection of its siRNA was performed twice in two-day intervals and in reverse fashion. Only a single transfection was done for HSET, however, as its near-complete KD using two transfections resulted in cell cycle arrest and significant cell death. This may be because HSET inhibits the ubiquitination-dependent degradation of the Inhibitor of Apoptosis family member Survivin, which has key roles in regulating cell division and inhibiting apoptosis [38]. Fig 3A and 3B show representative Western blots and quantitation of protein KDs for HeLa cells and MDA-MB-231 cells, respectively (see also S1 Table). Relative to cells receiving the control, non-targeting siRNA, Myo10 KD was in all cases close to complete (~96% depletion), and its KD altered HSET expression only slightly (~17% increase in HeLa and ~3% decrease in MDA-MB-231). Relative to cells receiving the control, non-targeting siRNA, HSET KD yielded ~86% depletion in HeLa cells and ~69% depletion in MDA-MB-231 cells, and in both cases its KD altered the expression of Myo10 only slightly (~1% decrease in HeLa and ~2% increase in MDA-MB-231). Finally, HSET depletion in double KD cells was slightly less efficient than in cells receiving only the HSET siRNA (~76% depletion in HeLa and ~63% depletion in MDA-MB-231). Importantly, staining control and KD HeLa cells at metaphase for Myo10 and HSET confirmed the localization data obtained using these antibodies, as the specific signals for both proteins were largely absent in their respective KD cells (S3 Fig).

To score defects in mitotic spindle organization, we fixed and stained control and KD cells 48 hours post transfection for γ-tubulin to label spindle poles, α-tubulin to label spindle microtubules, and DAPI to label chromosomes. Importantly, scoring was done using unsynchronized cells, as synchronization treatments such as low dose nocodazole can lead to the formation of multipolar spindles with abnormal centriole distributions due to cohesion fatigue [11]. Metaphase cells were optically sectioned in 0.25 µm intervals, and the sections used to count spindle pole numbers and to determine spindle and chromosome organization in 3D. Cells were scored as being either bipolar (this category included cells with just two spindle poles, one at each end of a normal bipolar spindle, and pseudo-bipolar cells, where one or both poles of a normal looking bipolar spindle were created by the clustering of supernumerary centrosomes; see S4 Fig A1-A4 for an example), semi-bipolar (cells possessing two major poles and a normal looking spindle, but also a minor pole in a different focal plane that contributed some microtubules to the spindle; see S4 Fig B1-B4 for an example), or multipolar (cells with more than two major spindle poles; see S4 Fig C1-C4 for an example).

**Fig 4:**
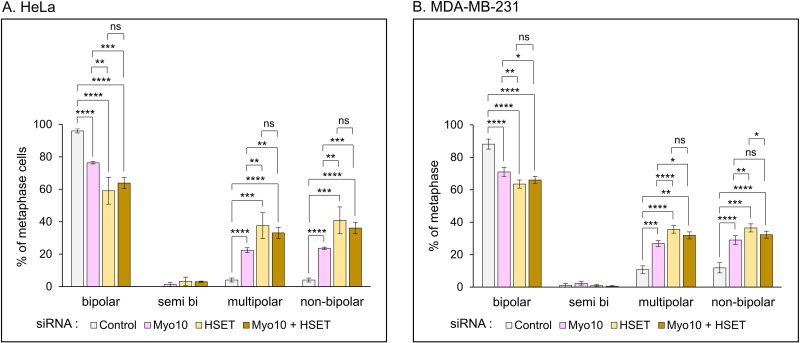
Knockdown of Myo10 and HSET individually increases the frequency of multipolar spindles in both HeLa and MDA-MB-231 cells, but their effects are not additive. (A) Percent of metaphase HeLa cells exhibiting bipolar, semi-polar, multipolar, or non-bipolar spindles in cells treated with control non-targeting siRNA (White), Myo10 siRNA (Pink), HSET siRNA (Tan), or both Myo10 and HSET siRNAs (Brown). (B) Same as (A) except for MDA-MB-231 cells. See also S2 Table.

Consistent with previous results [22], Myo10 KD HeLa cells exhibited a 20.5% decrease in the frequency of bipolar spindles, a 5.6-fold increase in the frequency of multipolar spindles, and a 5.9-fold increase in the frequency of non-bipolar spindles (semi-bipolar plus multipolar) relative to the non-targeting siRNA control cells (Fig 4A; S2 Table A). HSET KD resulted in even larger defects, with HSET KD HeLa cells exhibiting a 38.4% decrease in the frequency of bipolar spindles, a 9.4-fold increase in the frequency of multipolar spindles, and a 10.2-fold increase in the frequency of non-bipolar spindles (Fig 4A; S2 Table A; note that all of the HSET KD values were significantly larger than the corresponding Myo10 KD values). Similar results were obtained for Myo10 in MDA-MB-231 cells, where its KD resulted in a 19.5% decrease in the frequency of bipolar spindles, a 2.5-fold increase in the frequency of multipolar spindles, and a 2.5-fold increase in the frequency of non-bipolar spindles relative to the non-targeting siRNA control cells (Fig 4B; S2 Table B). Also as in HeLa cells, even larger defects were seen following HSET KD, with HSET KD MDA-MB-231 cells exhibiting a 28.0% decrease in the frequency of bipolar spindles, a 3.3-fold increase in the frequency of multipolar spindles, and a 3.1-fold increase in the frequency of non-bipolar spindles (Fig 4B; S2 Table B; note that all of the HSET KD values were significantly larger than the corresponding Myo10 KD values). Simultaneous KD of Myo10 and HSET in both cells types was not additive, however, as the decrease in spindle bipolarity and the increases in multipolar and non-bipolar spindles were not statistically larger in double KD cells than in HSET KD cells (although they were statistically larger than in Myo10 KD cells in all but one case) (Fig 4A and 4B; S2 Table). This lack of synergy was surprising given that these two motor proteins likely support SNCC via largely distinct mechanisms ([7]; see Discussion).

### An inability to cluster supernumerary centrosomes and PCM fragmentation drive the formation of multipolar spindles in HeLa cells depleted of Myo10 or HSET

Four distinct cellular defects can give rise to multipolar spindles [11]. Two of these defects, cytokinesis failure and centriole overduplication, create multipolar spindles because they generate cells that possess more than one G1 centrosome. The third and fourth defects, centriole disengagement and PCM fragmentation, create multipolar spindles because separated centrioles and acentriolar PCM fragments can both serve as additional MTOCs/poles. Importantly, HeLa cells depleted of Myo10, HSET or both do not exhibit significant increases relative to control cells in the average number of nuclei per cell (S5 Fig A; S3 Table A) or the average number of centrioles per cell (S5 Fig B; S3 Table B), arguing that cytokinesis failure and centriole overduplication do not contribute to their multipolar spindle phenotype. That leaves centriole disengagement and PCM fragmentation as possible contributors to a multipolar phenotype when these cells undergo mitosis possessing two normal centrosomes. Importantly, ~ 15% of HeLa cells possess supernumerary centrosomes at steady state [22,30]. For these cells, therefore, an additional contributor to a multipolar phenotype would be an inability to cluster their extra spindle poles (i.e., SNCC). Given this, subsequent experiments were designed to distinguish between centriole disengagement, PCM fragmentation, and an inability to cluster the extra poles in cells with supernumerary centrosomes (referred to in the figures as “de-clustering”) as causes of spindle multipolarity. To accomplish this, cells were stained for γ-tubulin, the centriole marker centrin-1, and DNA (DAPI), as this staining regimen allows one to distinguish between multipolar spindles created by each of these three possible defects (for representative examples of each defect, see Panels D, E and F in S4 Fig for images of unsynchronized, metaphase, Myo10-depleted HeLa cells stained in this way).

To quantitate the relative contributions made by centriole disengagement, PCM fragmentation, and centrosome de-clustering to creating multipolar spindles, we divided control and KD cells exhibiting multipolar spindles into two groups. One group contained the cells that possessed more than two normal centrosomes (i.e., centrosomes containing two centrioles). In this group, which we refer to in the figures as “>2 centrosomes”, defects in centriole disengagement, PCM fragmentation, and/or centrosome de-clustering could be responsible for their multipolar phenotype. The second group contained the cells that possessed two normal centrosomes. In this group, which we refer to in the figures as “2 centrosomes”, only defects in centriole disengagement and/or PCM fragmentation could be responsible for their multipolar phenotype.

**Fig 5:**
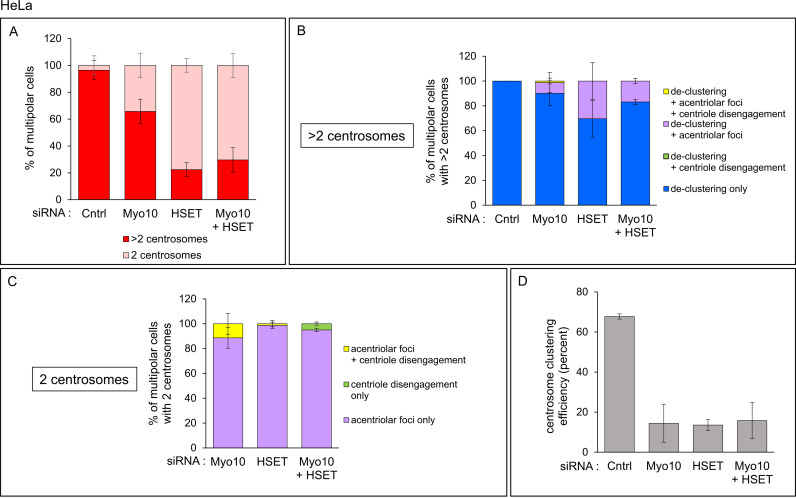
Causes of spindle multipolarity in HeLa cells depleted of Myo10, HSET or both. (A) Percent of multipolar HeLa cells treated with control non-targeting siRNA, Myo10 siRNA, HSET siRNA, or both Myo10 and HSET siRNAs that contained 2 centrosomes (Pink) or more than 2 centrosomes (Red). (B) Percent of multipolar HeLa cells with >2 centrosomes and treated with siRNAs as in (A) that exhibited centrosome de-clustering only (Blue), centrosome de-clustering plus acentriolar foci (Purple), centrosome de-clustering plus centriole disengagement (Green), or centrosome de-clustering plus acentriolar foci and centriole disengagement (Yellow). (C) Percent of multipolar HeLa cells with 2 centrosomes and treated with siRNAs as in (A) that exhibited acentriolar foci only (Purple), centriole disengagement only (Green) or acentriolar foci plus centriole disengagement (Yellow) (note that the control non-targeting siRNA was not scored because only ~3% of multipolar cells treated with this siRNA exhibited 2 centrosomes). (D) The efficiency of supernumerary centrosome clustering in HeLa cells for each siRNA (see text for details). All results are from four independent experiments. See also S4 Table.

Consistent with previous results [22], the vast majority (96.4 ± 7.1%) of control HeLa cells (i.e., cells treated with the non-targeting siRNA) that exhibited multipolar spindles contained more than 2 centrosomes (Fig 5A, Cntrl Red; S4 Table A). In all of these cells, every γ-tubulin spot contained at least two centrioles, indicating that centrosome de-clustering was solely responsible for their multipolar spindle phenotype (Fig 5B, Cntrl Blue; S4 Table B). For Myo10-depleted HeLa cells, about two thirds (65.7 ± 9.1%) of cells with multipolar spindles contained more than 2 centrosomes (Fig 5A, Myo10 Red; S4 Table A). In the vast majority of these cells (90.1 ± 10.0%), every γ-tubulin spot contained at least two centrioles, indicating that centrosome de-clustering was solely responsible for their multipolar spindle phenotype (Fig 5B, Myo10 Blue; S4 Table B). For the remaining 10% of Myo10-depleted cells with more than 2 centrosomes, scoring showed that de-clustering plus acentriolar foci generated by PCM fragmentation were largely responsible for their multipolar spindle phenotype (Fig 5B, Myo10 Purple; S4 Table B). Finally, for the 34.3 ± 9.1% of multipolar Myo10-depleted HeLa cells that contained 2 centrosomes (Fig 5A, Myo10 Pink; S4 Table A), scoring showed that acentriolar foci generated by PCM fragmentation was solely responsible for their multipolar spindle phenotype in 88.7 ± 8.4% of cases (Fig 5C, Myo10 Purple; S4 Table C). For the remaining ~11% of cases, acentriolar foci plus centriole disengagement were responsible for their multipolar spindle phenotype (Fig 5C, Myo10 Yellow; S4 Table C). Together, these results show that de-clustering is the major driver of multipolar spindles in Myo10-depleted HeLa cells possessing supernumerary centrosomes, and that PCM/spindle pole fragmentation is the major driver of multipolar spindles in Myo10-depleted HeLa cells lacking supernumerary centrosomes.

The results for HSET-depleted HeLa cells, while similar overall to the results for Myo10-depleted cells, differed is several ways. First, only about one quarter (22.4 ± 5.1%) of cells with multipolar spindles contained more than 2 centrosomes (Fig 5A, HSET Red; S4 Table A). Like Myo10-depleted cells, centrosome de-clustering was solely responsible for the multipolar spindle phenotype in the majority (69.6 ± 14.8%) of these cells (Fig 5B, HSET Blue; S4 Table B). For the remaining ~30% of HSET-depleted cells with more than 2 centrosomes, de-clustering plus acentriolar foci were responsible for their multipolar phenotype (Fig 5B, HSET Purple; S4 Table B). Finally, for the 77.6 ± 5.1% of multipolar HSET-depleted HeLa cells that contained 2 centrosomes (Fig 5A, HSET Pink; S4 Table A), acentriolar foci generated by PCM fragmentation was alone responsible for their multipolar spindle phenotype in 98.8 ± 2.5% of cases (Fig 5C, HSET Purple; S4 Table C). For the remaining ~1% of cases, acentriolar foci plus centriole disengagement were responsible (Fig 5C, HSET Yellow; S4 Table C). Together, these results show that, as in Myo10-depleted HeLa cells, de-clustering is the major driver of multipolar spindles in HSET-depleted HeLa cells possessing supernumerary centrosomes, and that PCM/spindle pole fragmentation is the major driver of multipolar spindles in HSET-depleted HeLa cells lacking supernumerary centrosomes.

### Like HeLa cells, centrosome de-clustering and PCM fragmentation contribute to the formation of multipolar spindles in MDA-MB-231 cells depleted of Myo10 or HSET, although the contribution made by PCM fragmentation is smaller than in HeLa cells

Similar to HeLa cells, MDA-MB-231 cells depleted of Myo10, HSET or both do not exhibit significant increases relative to control cells in the average number of nuclei per cell (S5 Fig C; S3 Table A) or the average number of centrioles per cell (S5 Figure D; S3 Table B), arguing that cytokinesis failure and centriole overduplication do not contribute to their multipolar spindle phenotype. That leaves centriole disengagement and PCM fragmentation as possible contributors to a multipolar phenotype when these cells undergo mitosis possessing 2 centrosomes. Importantly, ~ 35% of MDA-MB-231 cells possess supernumerary centrosomes at steady state [30]. For these cells, then, an additional contributor to a multipolar phenotype would be an inability to cluster their extra spindle poles (i.e., SNCC).

**Fig 6:**
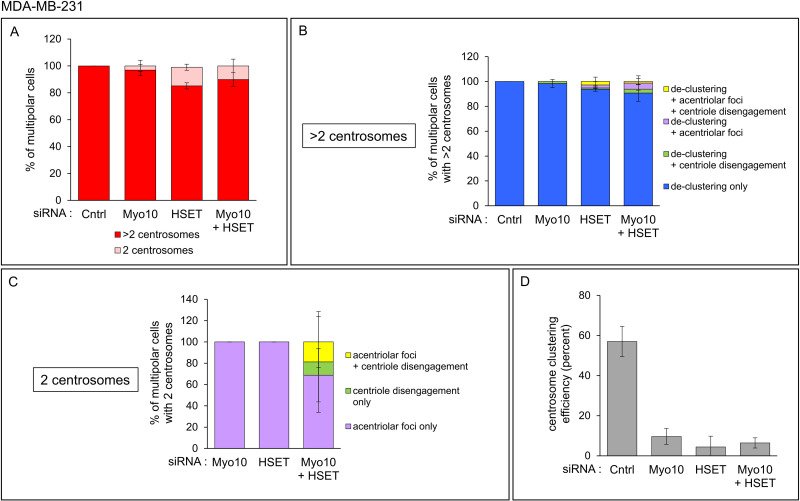
Causes of spindle multipolarity in MDA-MB-231 cells depleted of Myo10, HSET or both. (A) Percent of multipolar MDA-MB-231 cells treated with control non-targeting siRNA, Myo10 siRNA, HSET siRNA, or both Myo10 and HSET siRNAs that contained 2 centrosomes (Pink) or more than 2 centrosomes (Red). (B) Percent of multipolar MDA-MB-231 cells with >2 centrosomes and treated with siRNAs as in (A) that exhibited centrosome de-clustering only (Blue), centrosome de-clustering plus acentriolar foci (Purple), centrosome de-clustering plus centriole disengagement (Green), or centrosome de-clustering plus acentriolar foci and centriole disengagement (Yellow). (C) Percent of multipolar MDA-MB-231 cells with 2 centrosomes and treated with siRNAs as in (A) that exhibited acentriolar foci only (Purple), centriole disengagement only (Green) or acentriolar foci plus centriole disengagement (Yellow). (D) The efficiency of supernumerary centrosome clustering in MDA-MB-231 cells for each siRNA (see text for details). All results are from four independent experiments. See also S5 Table.

**Fig 7:**
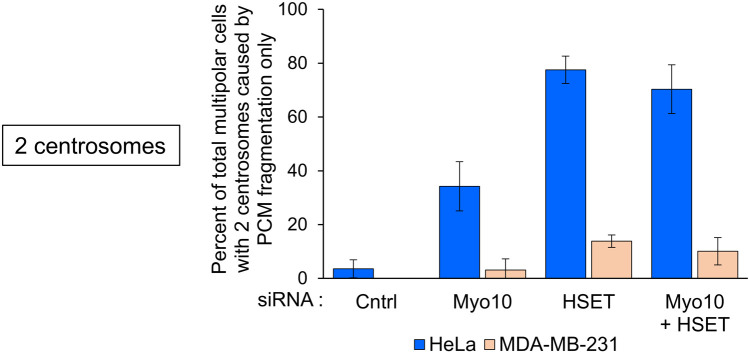
HeLa cells are more susceptible to PCM fragmentation than MDA-MB-231 cells following KD of either Myo10 or HSET. Shown is the percent of total multipolar HeLa and MDA-MB-231 cells treated with control non-targeting siRNA, Myo10 siRNA, HSET siRNA, or both Myo10 and HSET siRNAs where the multipolar phenotype was due solely to acentriolar foci created by PCM fragmentation. The results are from four independent experiments. See also S6 Table.

Consistent with previous work [30], 100% of control MDA-MB-231 cells (i.e., cells treated with the non-targeting siRNA) that exhibited multipolar spindles contained more than 2 centrosomes (Fig 6A, Cntrl Red; S5 Table A). While the results of Myo10 or HSET depletion in MDA-MB-231 cells were similar overall to those in HeLa cells, there were several differences, the most notable of which was that a much smaller fraction of multipolar Myo10-depleted and HSET-depleted MDA-MB-231 cells possessed only 2 centrosomes (3.1% and 13.9% for Myo10-depleted and HSET-depleted MDA-MB-231 cells, respectively, versus 34.3% and 77.6% for Myo10-depleted and HSET-depleted HeLa cells, respectively; Figs 5A and 6A; S4 Tables A and S5 Table A). Moreover, de-clustering alone was responsible for 98.4% and 93.8% of the multipolar phenotypes in Myo10-depleted and HSET-depleted MDA-MB-231 cells possessing more than 2 centrosomes, respectively, as compared to 90.1% and 69.6% in HeLa cells possessing more than 2 centrosomes (Figs 5B and 6B; S4 Table B and S5 Table B). In other words, for cells possessing more than 2 centrosomes, PCM fragmentation and centriole disengagement played a smaller role in creating a multipolar phenotype in MDA-MB-231 cells depleted of either Myo10 or HSET than in similarly treated HeLa cells. Stated another way, for cells possessing more than 2 centrosomes, HeLa cells are considerably more susceptible to PCM fragmentation than MDA-MB-231 cells. As with HeLa cells, the primary cause of spindle multipolarity in the small fraction of HSET KD MDA-MB-231 cells possessing only 2 centrosomes, and in the very small fraction of Myo10 KD MDA-MB-231 cells possessing only 2 centrosomes, was acentriolar foci caused by PCM fragmentation (Fig 6C; S5 Table C). That said, when the contribution of HeLa and MDA-MB-231 cells possessing only 2 centrosomes where PCM fragmentation was the sole cause of multipolar spindles is expressed as a percentage of total multipolar cells, it is clear that HeLa cells are much more susceptible to PCM fragmentation than MDA-MB-231 cells following the KD of either Myo10 or HSET (Fig 7 and S6 Table; see also S6 Fig for a representative example of multipolar spindles caused by PCM fragmentation in an HSET-depleted HeLa cell possessing only 2 centrosomes). In addition, HSET-depleted HeLa cells exhibit a more profound increase in the frequency of multipolar cells caused by PCM fragmentation than Myo10-depleted HeLa cells (Fig 7 and S6 Table).

### The contributions of de-clustering and PCM fragmentation to the formation of multipolar spindles in cells depleted of Myo10 and HSET simultaneously were not significantly different from that in cells depleted of HSET alone

Finally, the results for HeLa cells depleted of both Myo10 and HSET were essentially identical to the results for HeLa cells depleted of only HSET in both categories (i.e., 2 centrosomes and >2 centrosomes) (compare Myo10 + HSET to HSET in Fig 5A–5C; S4 Table). Consistently, the values for the efficiency of SNCC, which was calculated by dividing the number of cells exhibiting SNCC by the total number of cells with more than 2 centrosomes (yielding a value of 67.8 ± 1.3% for control cells), were very similar for all three knockdowns (14.4 ± 9.4% for Myo10, 13.6 ± 2.8% for HSET, and 15.8 ± 9.1% for Myo10 + HSET) (Fig 5D; S4 Table D). Similarly, the results for MDA-MB-231 cells depleted of both Myo10 and HSET were essentially identical to the results for MDA-MB-231 cells depleted of only HSET in both categories (compare Myo10 + HSET to HSET in Fig 6A–6C; S5 Table). Consistently, the values for the efficiency of centrosome clustering (which was 57.0 ± 7.5% for control MDA-MB-231 cells) were very similar for all three knockdowns (9.6 ± 4.0% for Myo10, 4.4 ± 5.4% for HSET, and 6.4 ± 2.5% for Myo10 + HSET) (Fig 6D; S5 Table D). Once again, this lack of synergy was surprising given that these two motor proteins likely support SNCC via largely distinct mechanisms.

### Both HSET knockdown and HSET inhibition using the drug CW069 alter the organization of metaphase HeLa cell retraction fibers

Kwon et al concluded that HSET’s contribution to SNCC was entirely actin independent based on their evidence that actin disassembly was not synergistic with Myo10 KD in terms of generating multipolar spindles (presumably because actin and Myo10 act in the same pathway), but was synergistic with HSET KD [12]. We found, on the other hand, that depletion of HSET in HeLa cells using siRNA disrupts the organization of the actin-rich retraction fibers that support cell adhesion during mitosis (compare the representative ventral confocal sections of a control siRNA-treated and an HSET siRNA-treated metaphase HeLa cell in S7 Fig, Panels A1-A4; see Panel B and S7 Table for the quantitation). Consistently, inhibition of HSET in HeLa cells using the anti-HSET drug CW069 ([32]; IC_50_ 75 µM) also disrupts the organization of metaphase retraction fibers (compare the representative ventral confocal sections of a control DMSO-treated and a CW069-treated metaphase HeLa cell in S7 Fig, Panels C1-C4). Both modes of HSET inhibition resulted in Myo10 tip-positive retraction fibers being shorter (see S7 Fig Panels D1 and D2, and S8 Table A for the quantitation) and less straight (S7 Fig Panels E1 and E2, and S8 Table B for the quantitation), although this later defect was more obvious in the CW069-treated cells (compare Panels E1 and E2; see also S8 Table B). CW069 also altered the actin cytoskeleton of interphase HeLa cells, with CW069-treated cells exhibiting relative to DMSO-treated cells more subnuclear stress fibers (S7 Fig; compare Box1 in F1/F2 to Box1 in G1/G2; see S7 Fig Panel H, and S9 Table A for the quantitation) and more peripheral stress fibers (S7 Fig; compare Box2 in F1/F2 to Box2 in G1/G2; see the arrow in G3 and S7 Fig Panel I, and S9 Table B for the quantitation). These observations are relevant for two reasons. First, they argue that HSET’s contribution to SNCC is not entirely actin independent, as previously thought [12] (although see Discussion). Second, because Myo10’s contribution to SNCC is via its ability to support retraction fiber-based cell adhesion during mitosis [22], depleting Myo10 in HSET KD cells may not be increasing the frequency of multipolar spindles at least in part because the KD of HSET alone alters the actin-rich structure through which Myo10 contributes.

### Depletion of either Myo10 or HSET reduces the content of Kizuna at metaphase spindle poles

As shown above, depletion of Myo10 or HSET in HeLa and MDA-MB-231 cells results in the PCM fragmentation-dependent formation of acentriolar spindle poles. Defects in spindle pole maturation can lead to defects in the structural integrity of poles that result in PCM fragmentation [11]. Such PCM fragmentation commonly occurs between prometaphase and metaphase when the chromosomal and spindle forces placed on the pole increase [11]. Consistently, PCM fragmentation in Myo10 knockout (KO) HeLa cells occurs primarily between prometaphase and metaphase, consistent with it being a force-dependent event [22]. That said, we also found that the recruitment of the pole maturation markers TPX2 and CDK5Rap2 is normal in Myo10 KO HeLa cells [22]. Moreover, we did not see Myo10 at spindle poles in our Halo-Myo10 knockin (KI) HeLa cell line [22]. Together, those results argued that PCM fragmentation in Myo10 depleted cells is not due to a general defect in pole maturation or to Myo10 having a direct role in maintaining pole stability (although see [39,40]).

Using HeLa cells, Oshimori et al [41] showed that the kinase Plk1 is required for the expansion of PCM during the process of spindle pole maturation, which occurs early in mitosis [42]. They also showed that Plk1 promotes spindle pole stability, and hence spindle bipolarity, by phosphorylating Threonine 379 in the ~ 75 kDa protein Kizuna, which accumulates at maturing spindle poles. Consistently, depletion of Kizuna resulted in PCM fragmentation at prometaphase and, as a consequence, the formation of multipolar spindles [41]. Other data in this study, together with results in a subsequent study by Thomas et al that focused on the regulation of Kizuna’s phosphorylation state by the combined actions of Plk1 and the phosphatase CDC25B [43], argue that keeping Threonine 379 in Kizuna phosphorylated through anaphase helps maintain pole stability by preventing the PCM fragmentation-dependent formation of multipolar spindles.

**Fig 8:**
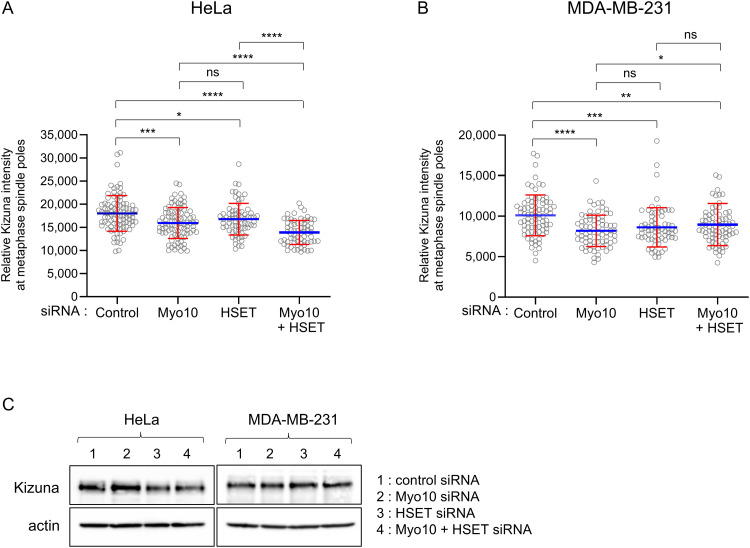
Depletion of either Myo10 or HSET reduces the spindle pole content of Kizuna. (A) Shown is the relative intensity of Kizuna staining at metaphase spindle poles in HeLa cells that had been treated with control non-targeting siRNA, Myo10 siRNA, HSET siRNA, or both Myo10 and HSET siRNAs and then stained for α-tubulin, Kizuna and DAPI. (B) Same as (A) except in MDA-MB-231 cells. Note that all of the samples were processed in parallel (i.e., fixing and staining) and imaged in parallel (i.e., identical imaging parameters), and that only bipolar cells were scored. See Materials and Methods for additional details. (C) Western blots showing the total cellular content of Kizuna in each of the samples. See also S11 Table.

Given the connection between Kizuna and PCM/pole stability, we decided to ask if the recruitment of Kizuna to spindle poles is defective in HeLa cells depleted of Myo10, which are especially prone to PCM fragmentation. As a first test, we stained WT HeLa and the Myo10 KO HeLa cell line KO-1 [22] at metaphase for Kizuna, α-tubulin, ƴ-tubulin, and DNA (DAPI). Consistent with previous studies [41,43], endogenous Kizuna was enriched at metaphase spindle poles in WT HeLa cells (S8 Fig A1-A4). While Kizuna was also present at spindle poles in KO-1 cells, its intensity relative to the intensity of the ƴ-tubulin signal appeared significantly lower than in WT HeLa (S8 Fig B1-B4). This was borne out by quantitation (S8 Fig C; S10 Table). To extend these results, we measured the pole content of Kizuna in HeLa cells and MDA-MB-231 cells depleted of Myo10, HSET or both using siRNA. All three knockdowns resulted in a significant reduction in Kizuna pole content relative to cells treated with the control non-targeting siRNA for both HeLa cells (Fig 8A; S11 Table) and MDA-MB-231 cells (Fig 8B; S11 Table). Western blotting indicated that these decreases could not be attributed to decreases in the cellular content of Kizuna (Fig 8C). While we do not know at present exactly why the depletion of Myo10 or HSET leads to significant reductions in Kizuna pole content, we can draw two partial conclusions from this data. The first is that it may explain at least in part the PCM fragmentation seen in cells depleted of Myo10 or HSET. The second relates to the fact that HeLa cells are much more susceptible to PCM fragmentation than MDA-MB-231 cells. Given that the percent decrease in Kizuna content was even higher in MDA-MB-231 cells than in HeLa cells (down 18.9% and 14.6% for Myo10 KD and HSET KD, respectively, in MDA-MB-231 versus down 11.5% and 6.9% for Myo10 KD and HSET KD, respectively, in HeLa; see S11 Table), we suggest that the increased susceptibility of HeLa cells to PCM fragmentation has more to do with the fact that they are more adherent than MDA-MB-231 cells (see Fig 1), as this should place additional strain on their spindle poles.

## Discussion

The survival of cells undergoing mitosis with more than two centrosomes depends on their ability to cluster their extra spindle poles so as to create a pseudo bipolar spindle, as this avoids a multipolar mitosis, the consequences of which are typically aneuploidy followed by cell senescence [6–10]. Given that the requirement for such SNCC is rare in normal cells but very common in both solid and hematological cancer cells, drugs that inhibit SNCC should selectively kill cancer cells. A seminal study by Kwon et al [12] identified two motor proteins that promote SNCC in MDA-MB-231 breast cancer cells: the minus end-directed kinesin 14 family member HSET/KIFC1 and the MyTH4/FERM myosin family member Myo10. That these two motor proteins are required for efficient SNCC makes sense as the pole focusing force created by HSET and the adhesion force created by Myo10’s FERM domain-dependent interaction with ECM-bound integrins at the tips of mitotic retraction fibers [22] would provide the spindle tension and the adhesion tension required for SNCC, respectively [6,7,9].

While Kwon et al did not test for cooperation between HSET and Myo10 in promoting SNCC, additive or synergistic effects would be expected given that these two motor proteins support distinct aspects of tension required for SNCC. Here we looked for such cooperation by depleting both proteins simultaneously in HeLa and MDA-MB-231 cells. To our surprise, the decrease in spindle bipolarity, and the increases in multipolar and non-bipolar spindles, were not statistically greater in double KD cells than in HSET KD cells (although they were statistically larger than in Myo10 KD cells in all but one case). One possible explanation for why HSET KD alone gave the maximal effect is our evidence here that HSET KD results in Myo10 tip-positive retraction fibers being shorter and less straight. Given that Myo10 promotes SNCC by promoting adhesion via these retraction fibers [22], we speculate that HSET KD alone gives the maximal effect because it is inhibiting not only HSET’s contribution to generating spindle tension but also Myo10’s contribution to generating adhesion tension.

Myo10 depletion in HeLa cells also results in PCM fragmentation, with the ensuing acentriolar spindle poles being the major driver of multipolar spindles in Myo10 KD HeLa cells lacking supernumerary centrosomes [22]. Given this, we also determined the contribution of PCM fragmentation to the formation of multipolar spindles in both HeLa and MDA-MB-231 cells following the KD of Myo10 alone, HSET alone, or both proteins. In every case, acentriolar foci generated by PCM fragmentation were almost entirely responsible for the formation of multipolar spindles in cells containing only 2 centrosomes. That said, HeLa and MDA-MB-231 cells differed dramatically in all three KD scenarios with regard to the percent of total multipolar cells due to PCM fragmentation only, with the values being 11.1, 5.6 and 7.0 times higher in Myo10 KD, HSET KD, and Myo10 + HSET KD HeLa cells, respectively, than in similarly treated MDA-MB-23 cells. One factor that may contribute to the heightened fragmentation of PCM in HeLa cells relative to MDA-MB-231 cells is their more adhesive nature relative to MDA-MB-231 cells (see Fig 1), as this may result in increased strain on HeLa cell spindle poles relative to MDA-MB-231 spindle poles during mitosis.

Kwon et al also concluded that HSET’s role in promoting SNCC is entirely actin independent [12]. We found, however, that HSET KD results in a large increase in the percent of metaphase HeLa cells with moderately to severely disorganized retraction fibers. Similar defects in retraction fiber organization were seen in metaphase HeLa cells that had been treated with the anti-HSET drug CW069 [32]. Given that retraction fibers are a major actin-based structure in mitotic cells, and that the adhesion they support plays an important role in SNCC [22], we suggest that HSET’s ability to promote SNCC may not be entirely actin independent. That said, we do not know at present how HSET promotes retraction fiber formation/organization. Moreover, the data we present falls well short of firmly establishing that HSET’s role in promoting SNCC depends on actin. We decided, nevertheless, to include this data to alert the many people interested in targeting HSET as a cancer therapeutic that HSET inhibition/loss alters the actin cytoskeleton. Finally, these results suggest that a better anti-HSET drug might be one that inhibits HSET’s pole focusing function without altering the actin cytoskeleton.

Defects in spindle pole maturation can lead to defects in the structural integrity of poles that result in PCM fragmentation [11]. We reported previously that the PCM fragmentation exhibited by Myo10 KO HeLa cells did not appear to be due to a general defect in pole maturation because the pole content of two spindle pole maturation markers (TPX2 and CDK5Rap2) was normal in these cells [22]. Here we found, however, that the pole content of Kizuna, which accumulates at maturing poles and is required for PCM stability [41,43], is significantly reduced in Myo10 KO HeLa cells, and in HeLa and MDA-MB-231 cells following the KD of Myo10, HSET or both. While HSET could in principle play a direct role in the recruitment of Kizuna because it localizes to forming spindle poles during prophase, Myo10 must play an indirect role because it is not present at spindle poles [22]. Nonetheless, the reduction in the pole content of Kizuna in cells depleted of Myo10, HSET or both may contribute to the PCM fragmentation exhibited by these cells. Of note, the decrease in the pole content of Kizuna is considerably greater in MDA-MB-231 cells (down 18.9% and 14.6% in Myo10 KD and HSET KD cells, respectively) than in HeLa cells (down 11.5% and 6.9% in Myo10 KD and HSET KD cells, respectively). This is notable because the percent of total multipolar cells due to PCM fragmentation is much higher for HeLa cells than for MDA-MB-231 cells. We suggest once again that this difference may be due to the more adhesive nature of HeLa cells, as this would likely result in increased strain on their spindle poles relative to MDA-MB-231 spindle poles during mitosis.

The mechanisms that drive SNCC represent excellent targets for cancer therapeutics because only transformed cells rely on SNCC for viability [6–10]. The identification in 2008 of HSET as one such target [12] has been supported by many subsequent studies showing that it is upregulated in numerous tumor types (e.g., breast, lung, pancreas, prostate, colon, bladder), that its upregulation correlates with tumor aggressiveness and poor prognosis, and that its knockdown in tumor cell models or primary tumor cells impairs SNCC [38,44–55]. In an effort to develop a therapeutic, Watts et al used chemogenomics, convergent syntheses and other approaches to generate the synthetic compound CW069, which they showed inhibits HSET *in vitro* in allosteric fashion [32]. Importantly, CW069 treatment induced multipolar mitoses only in cells containing supernumerary centrosomes. That said, because the authors did not use a centriole marker like centrin, their data did not parse out the relative contributions of defective SNCC and PCM fragmentation to the multipolar phenotype exhibited by CW069 treated cells. The authors also noted that CW069 exhibited anti-proliferative effects even in cancer cell lines with normal centrosome number, indicating that its growth inhibitory effects cannot be due solely to multipolar mitosis-induced aneuploidy. Finally, three additional inhibitors of HSET have been identified (AZ82 [56–58]; SR31527 [59], and KAA [50]), and others may be on the way [60,61].

The results of Kwon et al [12], together with our previous results [22] and the results presented here, argue that an inhibitor of Myo10 might also be effective as a cancer therapeutic. Moreover, several recent studies have lent additional support for this idea. First, the depletion of Myo10 in a mouse model of melanoma was shown to reduce melanoma development and metastasis and to extend medial survival time [62]. Second, the lifespan of mice with glioblastoma was shown to be extended significantly on a Myo10 KO background [63]. Third, breast cancer progression in mice was shown to be inhibited by Myo10 depletion and accelerated by Myo10 overexpression [40]. More generally, many cancer cell types exhibit elevated levels of Myo10, which may support their growth by promoting SNCC [64–66]. These and other studies showing that Myo10-positive filopodia promote the migration of metastatic cancer cells [65], together with the data presented here, provide strong justification for performing screens to identify inhibitors of Myo10 as possible cancer therapeutics. Finally, while we did not see additive effects in either HeLa or MDA-MB-231 cells when we depleted HSET and Myo10 simultaneously using siRNA, it is certainly possible that compounds that inhibit these two motor proteins might act in an additive or synergistic fashion in the context of both cell models and tumors *in situ*, especially if the anti-HSET drug does not alter the actin cytoskeleton.

## Supporting information

S1 Fig The localization of endogenous HSET in interphase and mitotic MDA-MB-231 cells is similar to that in HeLa cells. Representative images of MDA-MB-231 cells stained for Myo10, HSET and DNA (DAPI) at interphase (A1-A4), prophase (B1-B5), metaphase (C1-C5), and late anaphase (D1-D5). Shown are middle sections and bottom sections. The white arrows in B3 mark the positions of the two spindle poles. All mag bars are 10 µm.
(TIF)

S2 Fig Myo10 and HSET expression levels in HeLa and MDA-MB-231 cells. (A) Representative Western blot of whole cell extracts of HeLa and MDA-MB-231 cells probed with antibodies against Myo10, HSET and actin. (B) Means and standard deviations of expression levels for Myo10 and HSET from three separate experiments. The densitometry data was normalized using the actin band intensities, and the mean HeLa cell expression level for both proteins was set to a value of 1.0. The results show that Myo10 expression in MDA-MB-231 is 87.8 ± 3.7% that in HeLa, while HSET expression in MDA-MB-231 is 3.0 ± 0.2 fold higher than in HeLa.
(TIF)

S3 Fig Staining of HeLa KD cells at metaphase for Myo10 and HSET confirmed the localization data obtained using the antibodies. Representative images of metaphase HeLa cells that had been treated with control non-targeting siRNA, Myo10 siRNA, HSET siRNA, or both Myo10 and HSET siRNAs and stained for Myo10 and HSET. Note that the strong signal for Myo10 at the tips of retraction fibers in cells that did not receive Myo10 siRNA (A4 and C4) is largely absent in cells that received the Myo10 siRNA (B4 and D4) (see also [22]). Similarly, the strong signal for HSET on the spindle in cells that did not receive HSET siRNA (A3 and B3) is largely absent in cells that received the HSET siRNA (C3 and D3). All mag bars are 10 µm.
(TIF)

S4 Fig Representative examples of spindle phenotypes, centriole disengagement, PCM fragmentation, and de-clustering. (A-C) Representative images of metaphase HeLa cells stained for α-tubulin, ƴ-tubulin, and DNA (DAPI) that show examples of a bipolar spindle (A1-A4), a semi-polar spindle (B1-B4) and a multipolar spindle (C1-C4). (D-F) Representative images of multipolar Myo10 KD HeLa cells at metaphase stained for centrin-1, ƴ-tubulin, and DNA (DAPI) that show examples of centriole disengagement (D), PCM fragmentation (E), and de-clustering (F). All mag bars are 5 µm.
(TIF)

S5 Fig Numbers of nuclei and centrioles per cell. (A) Average number of nuclei per cell for HeLa cells treated with control non-targeting siRNA, Myo10 siRNA, HSET siRNA, or both Myo10 and HSET siRNAs determined by imaging cells stained for F-actin (Phalloidin) and DNA (DAPI). (B) Average number of centrioles per cell for HeLa cells treated with control non-targeting siRNA, Myo10 siRNA, HSET siRNA, or both Myo10 and HSET siRNAs determined by imaging cells stained for centrin-1, ƴ-tubulin and DNA (DAPI) (only cells with one nucleus were scored). (C) Same as (A) except for MDA-MB-231 cells. (D) Same as (B) except for MDA-MB-231 cells. Note that the values in (D) for HSET KD and combined KD of Myo10 KD and HSET, while significantly different from the control, represent decreases in centriole number, not increases (these decreases may be due to the anti-proliferative effects of HSET KD). The results are from three independent experiments. See also S3 Table.
(TIF)

S6 Fig Representative example of multipolar spindles caused by PCM fragmentation in an HSET-depleted HeLa cell. Shown are the centrin-1, α-tubulin and ƴ-tubulin signals for six α-tubulin- and ƴ-tubulin-positive spindle poles seen in the Z-Stack overlay of an HSET siRNA treated HeLa cell. Only two of the six α-tubulin- and ƴ-tubulin-positive spindle poles have a centrin-1 signal, indicating that the other four spindle poles were created by PCM fragmentation. Mag bar is 5 µm.
(TIF)

S7 Fig HSET knockdown and HSET inhibition using the drug CW069 alter the organization of metaphase HeLa cell retraction fibers. (A1-A4) Shown are representative ventral confocal sections of a control siRNA-treated metaphase HeLa cell (A1 and A2) and an HSET siRNA-treated metaphase HeLa cell (A3 and A4) that were stained for Myo10, actin (Phalloidin) and DNA (DAPI). (B) Percent of metaphase HeLa cells exhibiting organized, moderately disorganized, or severely disorganized retraction fibers (RFs) in HeLa cells treated with non-targeting siRNA (Cntrl) or HSET siRNA along with representative images. (C1-C4) Shown are representative ventral confocal sections of a control DMSO-treated metaphase HeLa cell (C1 and C2) and a CW069-treated metaphase HeLa cell (C3 and C4) stained as in (A). (D1 and D2) RF lengths (in µm) for HeLa cells treated with non-targeting siRNA (Cntrl) or HSET siRNA (D1) and for HeLa cells treated with DMSO or CW069 (D2) (100 RFs from 10 representative cells each, none with severely disorganized RFs, were scored). (E1 and E2) RF straightness (a value of 1.0 is perfectly straight) for HeLa cells treated with non-targeting siRNA (Cntrl) or HSET siRNA (E1) and for HeLa cells treated with DMSO or CW069 (E2) (100 RFs from 10 representative cells each, none with severely disorganized RFs, were scored). (F and G) Shown are representative ventral confocal sections of a control DMSO-treated interphase HeLa cell (F1-F3) and a CW069-treated interphase HeLa cell (G1-G3) stained as in (A). (H) Total intensities (in arbitrary units) of fluorescent Phalloidin stained subnuclear stress fibers (SFs) in HeLa cells treated with DMSO or CW069 (from 66 cells for DMSO and 57 cells for CW069 over three separate experiments). (I) Distances (in µm) between peripheral SFs and the cell’s leading edge for HeLa cells treated with DMSO or CW069 (from 33 cells for DMSO and 41 cells for CW069 over three separate experiments). See the text and Methods for additional details, and S7-S9 Tables for quantitation. All mag bars are 10 µm.
(TIF)

S8 Fig Myo10 KO-1 HeLa cells exhibit a reduction in Kizuna content at metaphase spindle poles. (A1-A4) Shown is a representative equatorial confocal section of a WT HeLa cell at metaphase that was stained for α-tubulin, Kizuna and DAPI. (B1-B4) Shown is a representative equatorial confocal section of a Myo10 KO-1 HeLa cell [22] at metaphase stained as in (A) and imaged using identical imaging parameters. (C) Relative Kizuna intensity at metaphase spindle poles (see also S10 Table; note that only bipolar cells were scored). All mag bars are 5 µm.
(TIF)

S1 Table SiRNA-mediated knockdown efficiencies. This is the statistic outcomes corresponding to Figure 3. HeLa from 4 independent experiments, MDA-MB-231 from 3 independent experiments.
(TIF)

S2 Table Spindle type frequencies. This is the statistic outcomes corresponding to Figure 4. A. HeLa: from 4 independent experiments, B. MDA-MB-231: from 4 independent experiments.
(TIF)

S3 Table Number of nuclei and centrioles per cell. This is the statistic outcomes corresponding to S5 Figure. A. Number of nuclei/ cell, B. Number of centrioles/ cell.
(TIF)

S4 Table Causes of spindle multipolarity in HeLa KD cells. This is the statistic outcomes corresponding to Figure 5. A. The percentage of multipolar cells: from 4 independent experiments, B. The percentage of multipolar cells with >2 centrosomes: from 4 independent experiments, C. The percentage of multipolar cells with 2 centrosomes: from 4 independent experiments, D. Centrosome clustering efficiency: from 4 independent experiments.
(TIF)

S5 Table Causes of spindle multipolarity in MDA-MB-231 KD cells. This is the statistic outcomes corresponding to Figure 6. A. The percentage of multipolar cells: from 4 independent experiments, B. The percentage of multipolar cells with >2 centrosomes: from 4 independent experiments, C. The percentage of multipolar cells with 2 centrosomes: from 4 independent experiments, D. Centrosome clustering efficiency: from 4 independent experiments.
(TIF)

S6 Table The percentage of total multipolar cells with 2 centrosomes caused by PCM fragmentation only. This is the statistic outcomes corresponding to Figure 7. The results came from 4 independent experiments for both cell lines.
(TIF)

S7 Table Retraction fiber morphology. This is the statistic outcomes corresponding to S7 Figure. The results came from 3 independent experiments in HeLa.
(TIF)

S8 Table Retraction fiber organization. A. Retraction fiber length, the statistic outcomes corresponding to S7 Figure D1 and D2, B. Retraction fiber straightness, the statistic outcomes corresponding to S7 Figure E1 and E2.
(TIF)

S9 Table Actin organization in interphase cells. A. Actin intensity under the nucleus, the statistic outcomes corresponding to S7 Figure H, B. Distance between peripheral SFs and leading edge, the statistic outcomes corresponding to S7 Figure I.
(TIF)

S10 Table Content of Kizuna at metaphase spindle poles in HeLa Myo10 KO-1. This is the statistic outcomes corresponding to S8 Figure.
(TIF)

S11 Table Content of Kizuna at metaphase spindle poles. This is the statistic outcomes corresponding to Figure 8.
(TIF)
