# Supplementary figures and images for: Two cancer cell lines utilize Myosin 10 and the kinesin HSET differentially to maintain mitotic spindle bipolarity
Source: PLoS One. 2025 May 29;20(5):e0325016. doi: 10.1371/journal.pone.0325016 (PMC12121739; doi:10.1371/journal.pone.0325016)

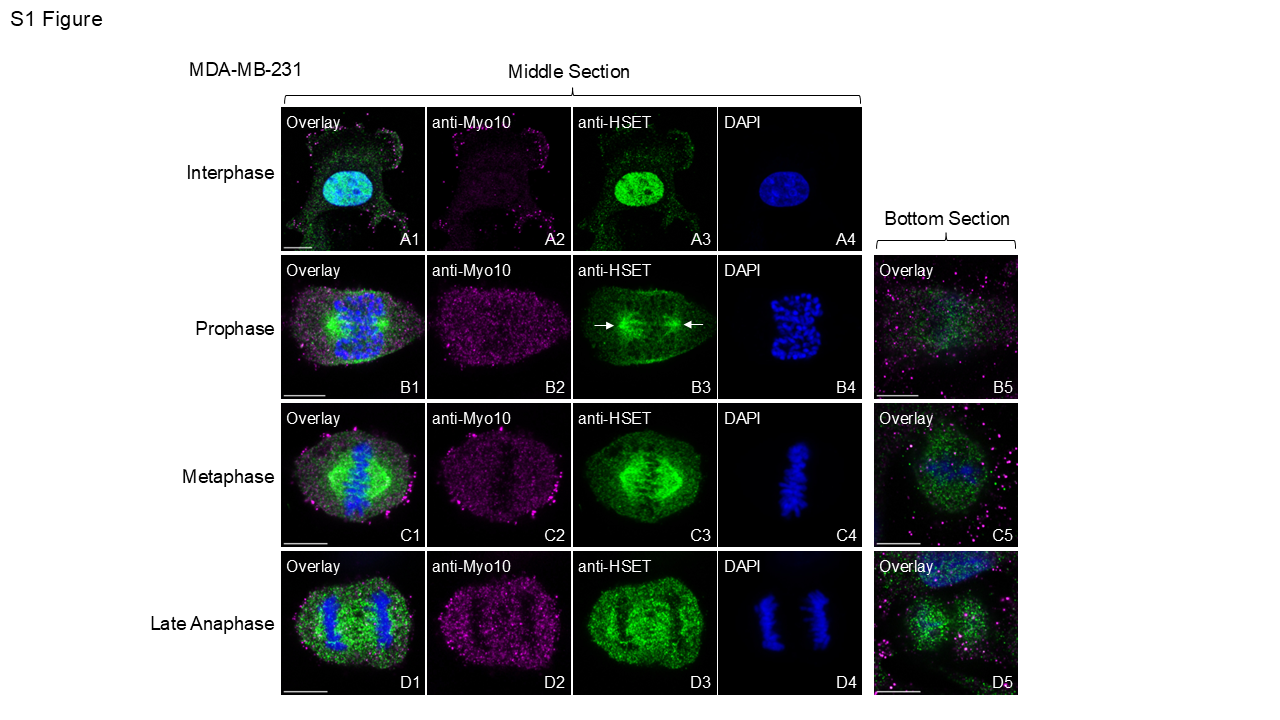

Supplement: S1 Fig — Representative images of MDA-MB-231 cells stained for Myo10, HSET and DNA (DAPI) at interphase (A1-A4), prophase (B1-B5), metaphase (C1-C5), and late anaphase (D1-D5). Shown are middle sections and bottom sections. The white arrows in B3 mark the positions of the two spindle poles. All mag bars are 10 µm. (TIF) [file pone.0325016.s001.tif]

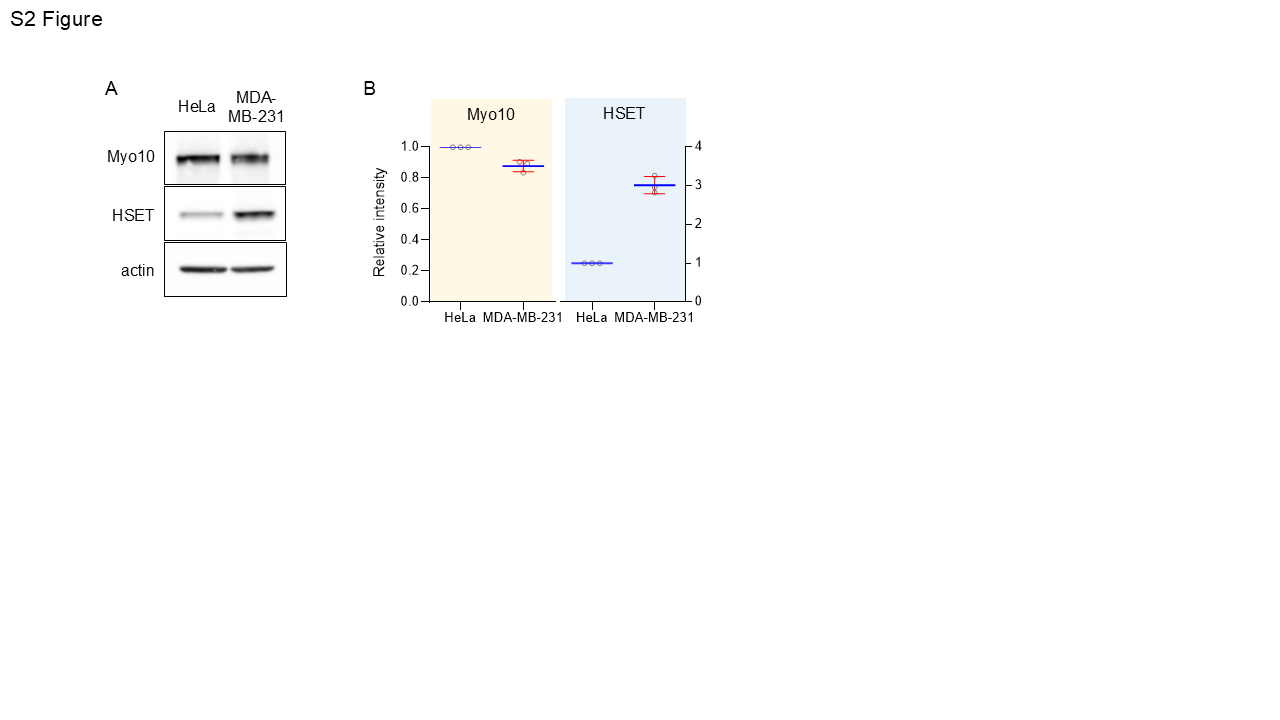

Supplement: S2 Fig — (A) Representative Western blot of whole cell extracts of HeLa and MDA-MB-231 cells probed with antibodies against Myo10, HSET and actin. (B) Means and standard deviations of expression levels for Myo10 and HSET from three separate experiments. The densitometry data was normalized using the actin band intensities, and the mean HeLa cell expression level for both proteins was set to a value of 1.0. The results show that Myo10 expression in MDA-MB-231 is 87.8 ± 3.7% that in HeLa, while HSET expression in MDA-MB-231 is 3.0 ± 0.2 fold higher than in HeLa. (TIF) [file pone.0325016.s002.tif]

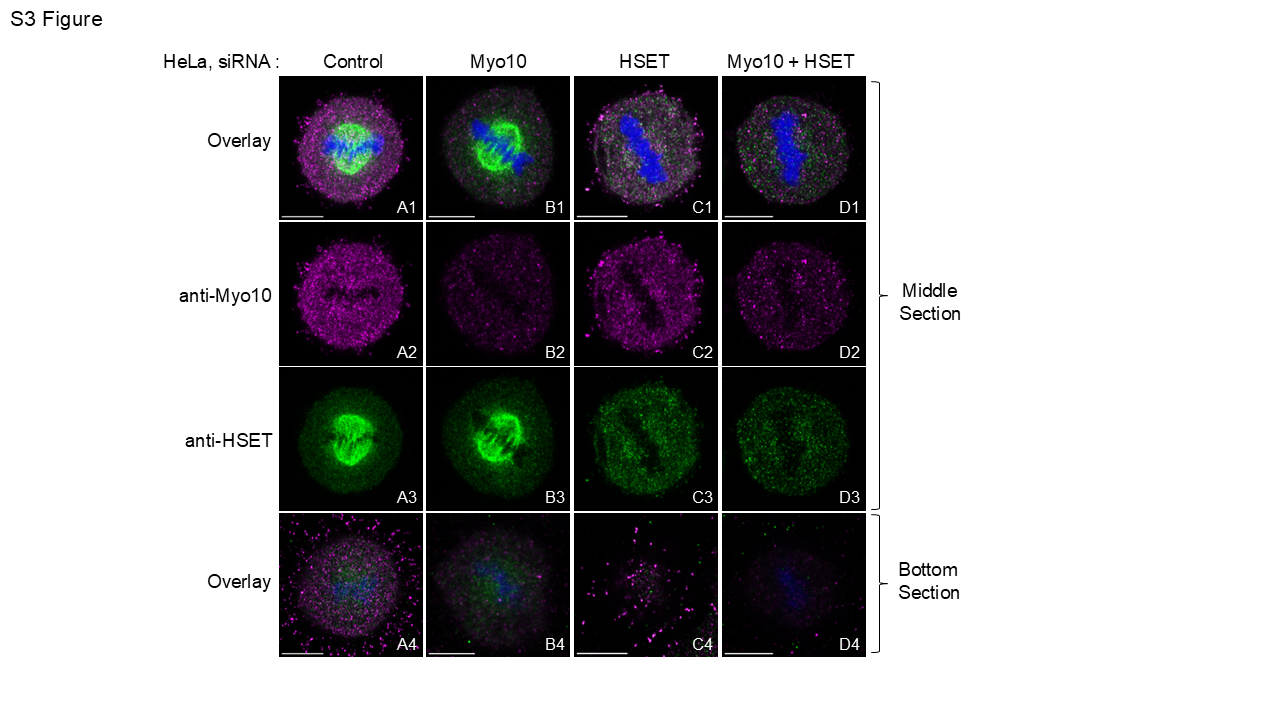

Supplement: S3 Fig — Representative images of metaphase HeLa cells that had been treated with control non-targeting siRNA, Myo10 siRNA, HSET siRNA, or both Myo10 and HSET siRNAs and stained for Myo10 and HSET. Note that the strong signal for Myo10 at the tips of retraction fibers in cells that did not receive Myo10 siRNA (A4 and C4) is largely absent in cells that received the Myo10 siRNA (B4 and D4) (see also [22]). Similarly, the strong signal for HSET on the spindle in cells that did not receive HSET siRNA (A3 and B3) is largely absent in cells that received the HSET siRNA (C3 and D3). All mag bars are 10 µm. (TIF) [file pone.0325016.s003.tif]

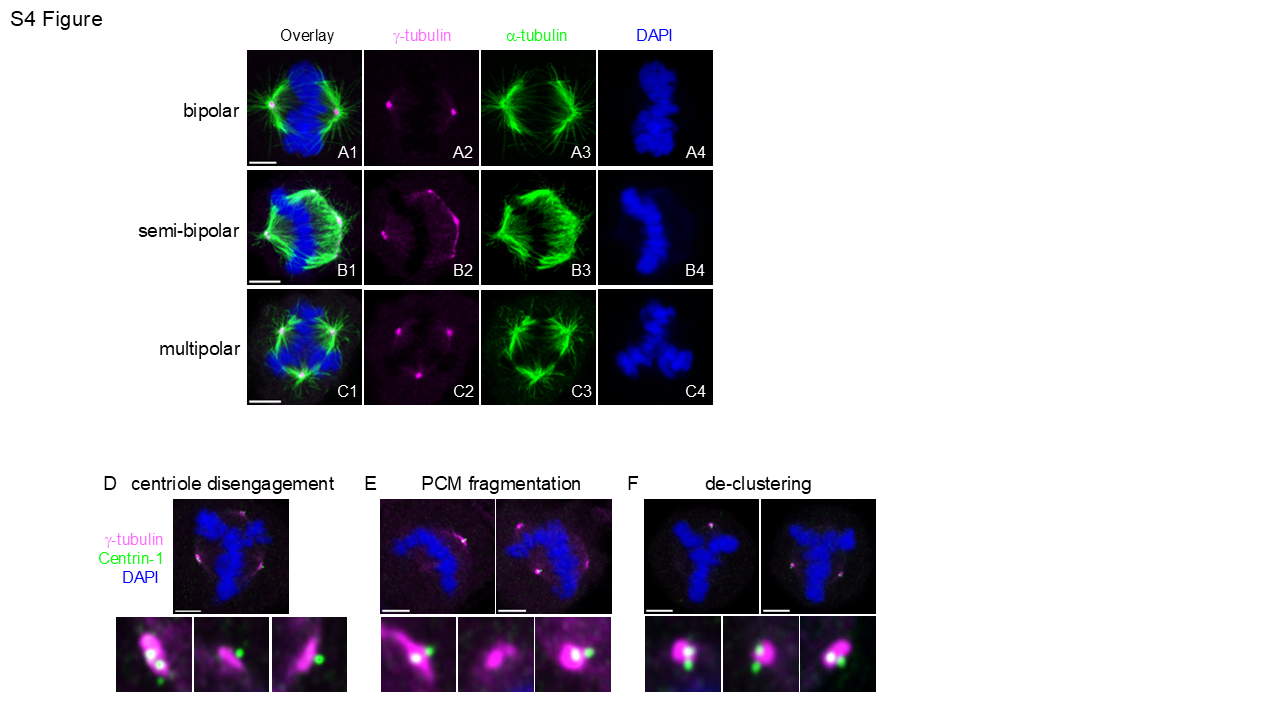

Supplement: S4 Fig — (A-C) Representative images of metaphase HeLa cells stained for α-tubulin, ƴ-tubulin, and DNA (DAPI) that show examples of a bipolar spindle (A1-A4), a semi-polar spindle (B1-B4) and a multipolar spindle (C1-C4). (D-F) Representative images of multipolar Myo10 KD HeLa cells at metaphase stained for centrin-1, ƴ-tubulin, and DNA (DAPI) that show examples of centriole disengagement (D), PCM fragmentation (E), and de-clustering (F). All mag bars are 5 µm. (TIF) [file pone.0325016.s004.tif]

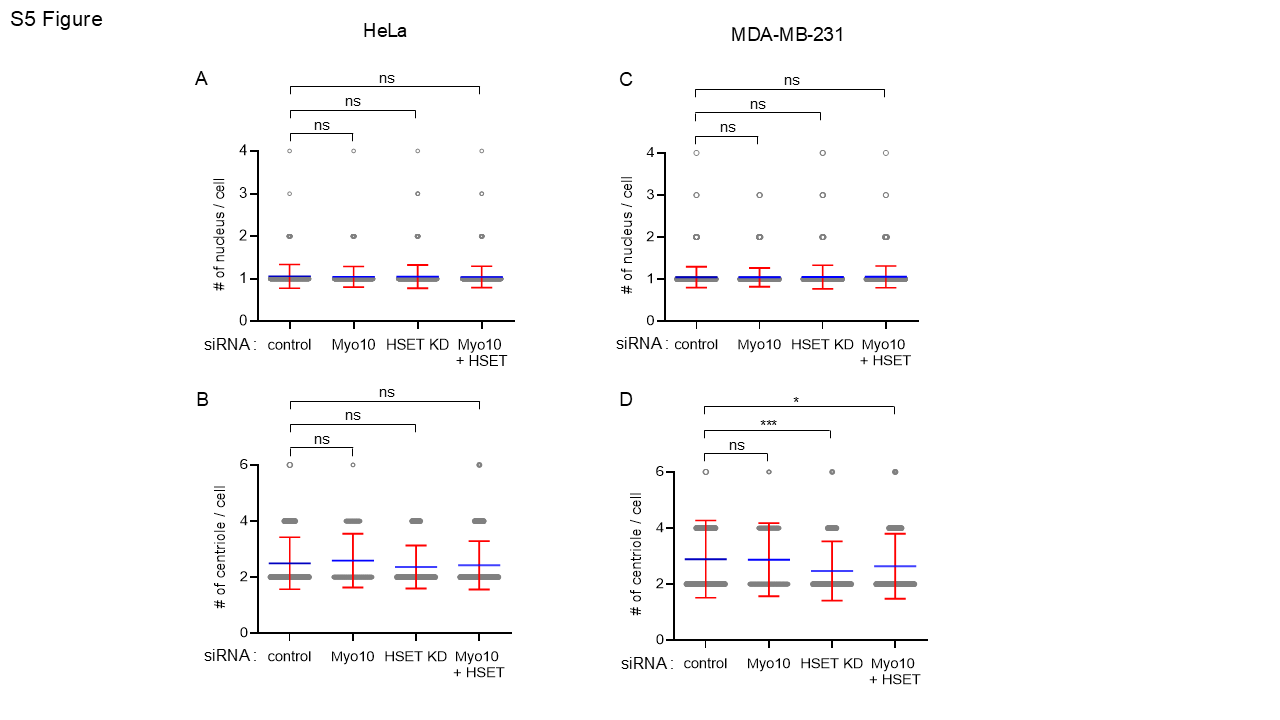

Supplement: S5 Fig — (A) Average number of nuclei per cell for HeLa cells treated with control non-targeting siRNA, Myo10 siRNA, HSET siRNA, or both Myo10 and HSET siRNAs determined by imaging cells stained for F-actin (Phalloidin) and DNA (DAPI). (B) Average number of centrioles per cell for HeLa cells treated with control non-targeting siRNA, Myo10 siRNA, HSET siRNA, or both Myo10 and HSET siRNAs determined by imaging cells stained for centrin-1, ƴ-tubulin and DNA (DAPI) (only cells with one nucleus were scored). (C) Same as (A) except for MDA-MB-231 cells. (D) Same as (B) except for MDA-MB-231 cells. Note that the values in (D) for HSET KD and combined KD of Myo10 KD and HSET, while significantly different from the control, represent decreases in centriole number, not increases (these decreases may be due to the anti-proliferative effects of HSET KD). The results are from three independent experiments. See also S3 Table. (TIF) [file pone.0325016.s005.tif]

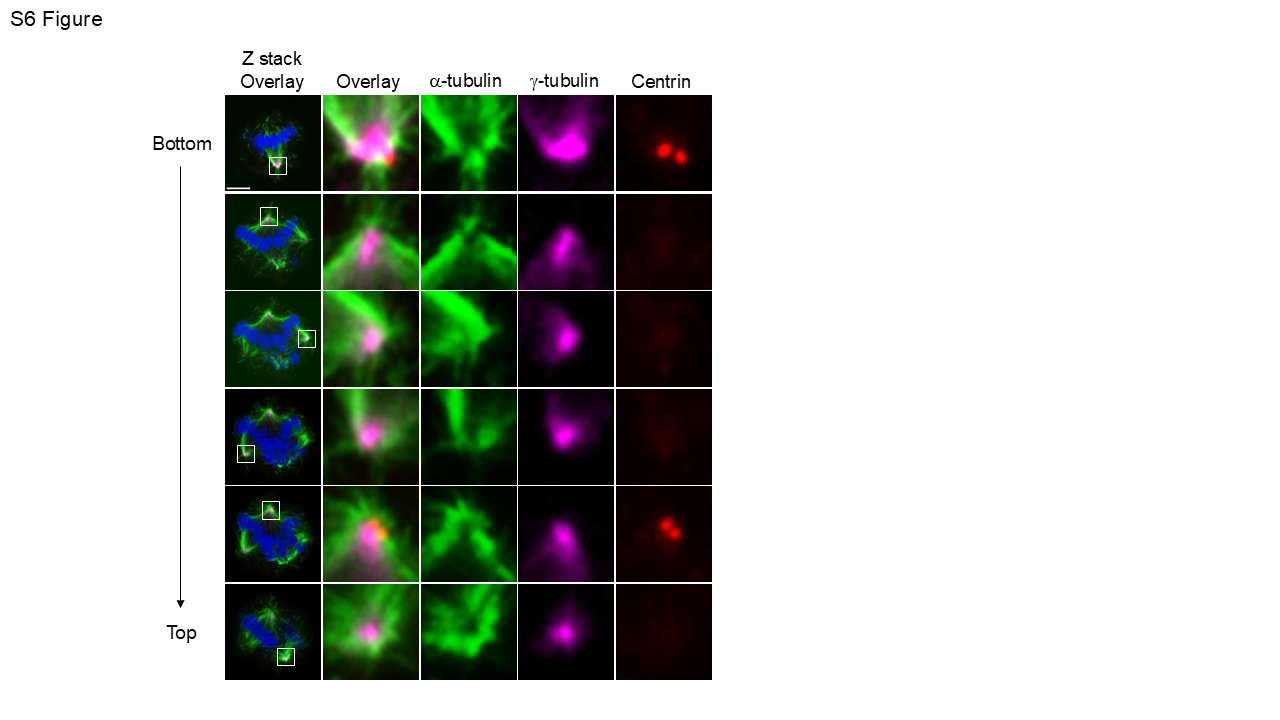

Supplement: S6 Fig — Shown are the centrin-1, α-tubulin and ƴ-tubulin signals for six α-tubulin- and ƴ-tubulin-positive spindle poles seen in the Z-Stack overlay of an HSET siRNA treated HeLa cell. Only two of the six α-tubulin- and ƴ-tubulin-positive spindle poles have a centrin-1 signal, indicating that the other four spindle poles were created by PCM fragmentation. Mag bar is 5 µm. (TIF) [file pone.0325016.s006.tif]

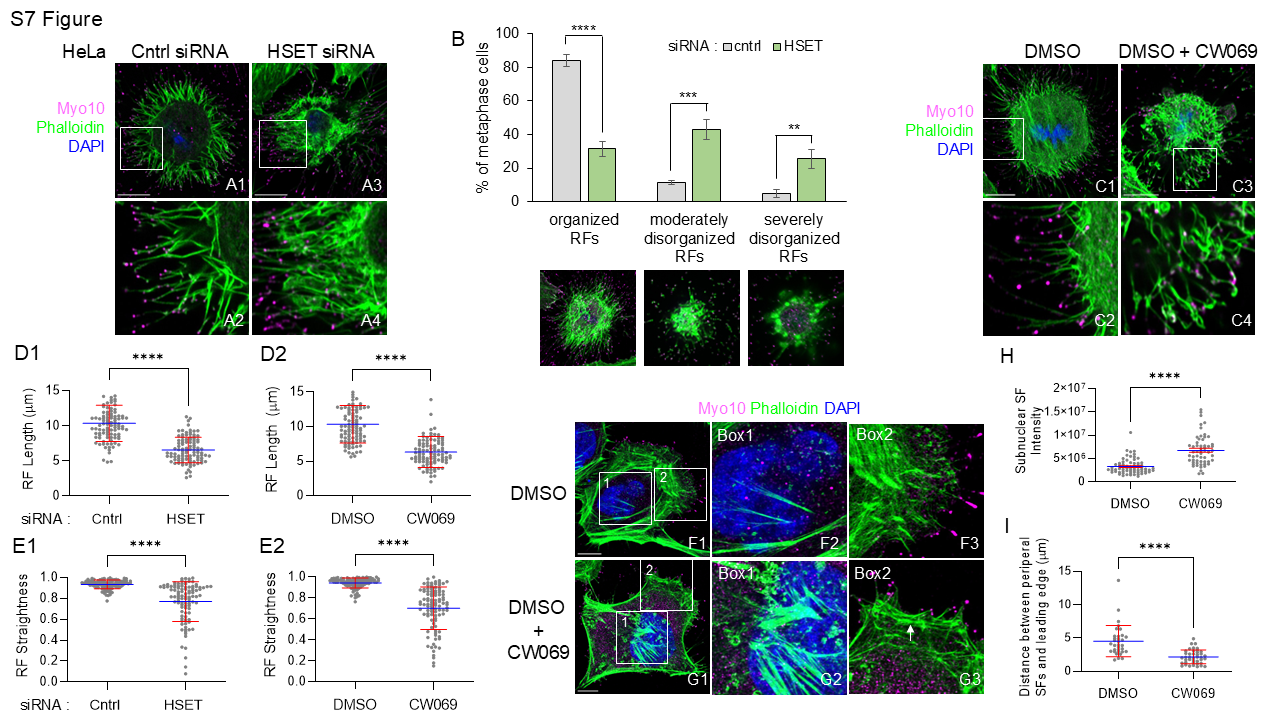

Supplement: S7 Fig — (A1-A4) Shown are representative ventral confocal sections of a control siRNA-treated metaphase HeLa cell (A1 and A2) and an HSET siRNA-treated metaphase HeLa cell (A3 and A4) that were stained for Myo10, actin (Phalloidin) and DNA (DAPI). (B) Percent of metaphase HeLa cells exhibiting organized, moderately disorganized, or severely disorganized retraction fibers (RFs) in HeLa cells treated with non-targeting siRNA (Cntrl) or HSET siRNA along with representative images. (C1-C4) Shown are representative ventral confocal sections of a control DMSO-treated metaphase HeLa cell (C1 and C2) and a CW069-treated metaphase HeLa cell (C3 and C4) stained as in (A). (D1 and D2) RF lengths (in µm) for HeLa cells treated with non-targeting siRNA (Cntrl) or HSET siRNA (D1) and for HeLa cells treated with DMSO or CW069 (D2) (100 RFs from 10 representative cells each, none with severely disorganized RFs, were scored). (E1 and E2) RF straightness (a value of 1.0 is perfectly straight) for HeLa cells treated with non-targeting siRNA (Cntrl) or HSET siRNA (E1) and for HeLa cells treated with DMSO or CW069 (E2) (100 RFs from 10 representative cells each, none with severely disorganized RFs, were scored). (F and G) Shown are representative ventral confocal sections of a control DMSO-treated interphase HeLa cell (F1-F3) and a CW069-treated interphase HeLa cell (G1-G3) stained as in (A). (H) Total intensities (in arbitrary units) of fluorescent Phalloidin stained subnuclear stress fibers (SFs) in HeLa cells treated with DMSO or CW069 (from 66 cells for DMSO and 57 cells for CW069 over three separate experiments). (I) Distances (in µm) between peripheral SFs and the cell’s leading edge for HeLa cells treated with DMSO or CW069 (from 33 cells for DMSO and 41 cells for CW069 over three separate experiments). See the text and Methods for additional details, and S7-S9 Tables for quantitation. All mag bars are 10 µm. (TIF) [file pone.0325016.s007.tif]

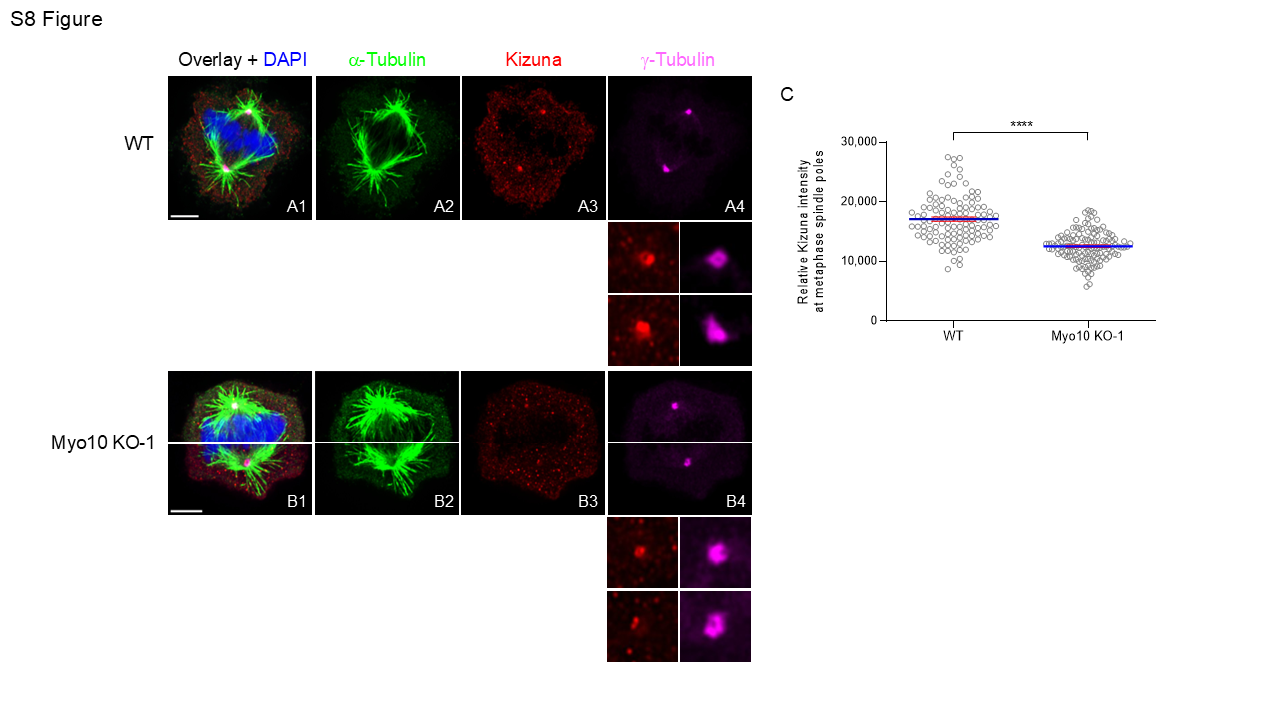

Supplement: S8 Fig — (A1-A4) Shown is a representative equatorial confocal section of a WT HeLa cell at metaphase that was stained for α-tubulin, Kizuna and DAPI. (B1-B4) Shown is a representative equatorial confocal section of a Myo10 KO-1 HeLa cell [22] at metaphase stained as in (A) and imaged using identical imaging parameters. (C) Relative Kizuna intensity at metaphase spindle poles (see also S10 Table; note that only bipolar cells were scored). All mag bars are 5 µm. (TIF) [file pone.0325016.s008.tif]

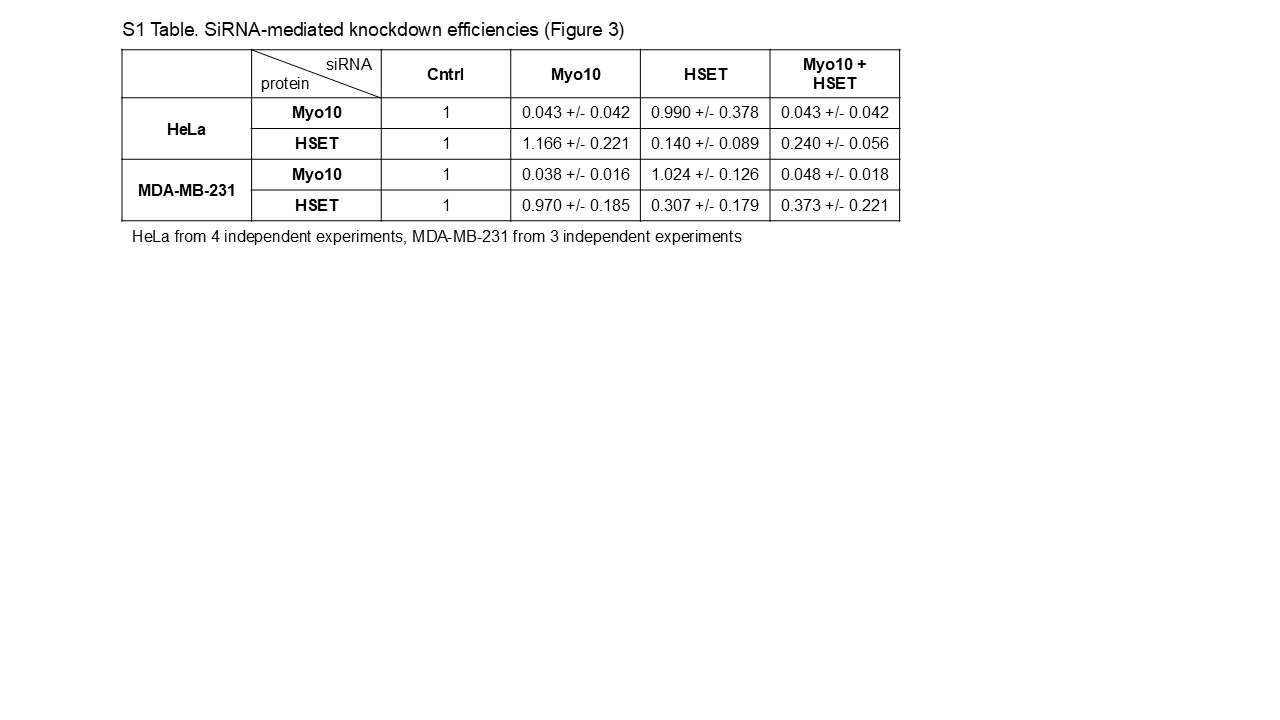

Supplement: S1 Table — This is the statistic outcomes corresponding to Figure 3. HeLa from 4 independent experiments, MDA-MB-231 from 3 independent experiments. (TIF) [file pone.0325016.s009.tif]

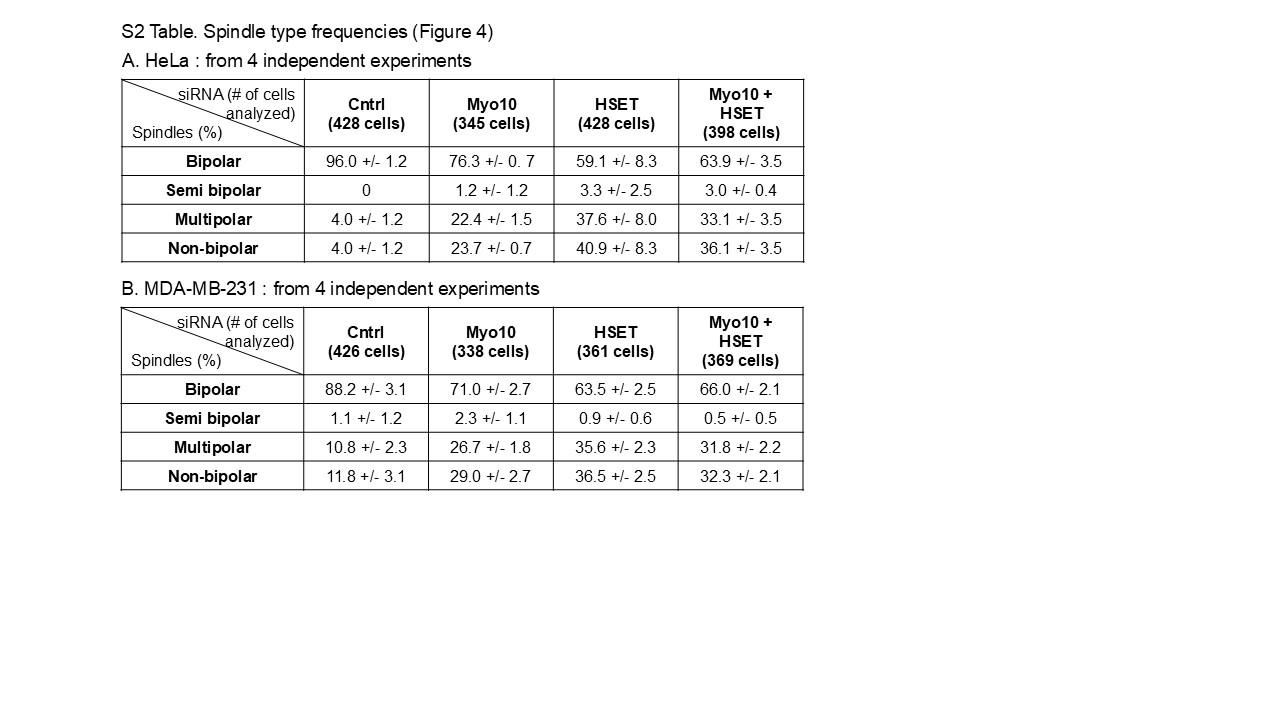

Supplement: S2 Table — This is the statistic outcomes corresponding to Figure 4. A. HeLa: from 4 independent experiments, B. MDA-MB-231: from 4 independent experiments. (TIF) [file pone.0325016.s010.tif]

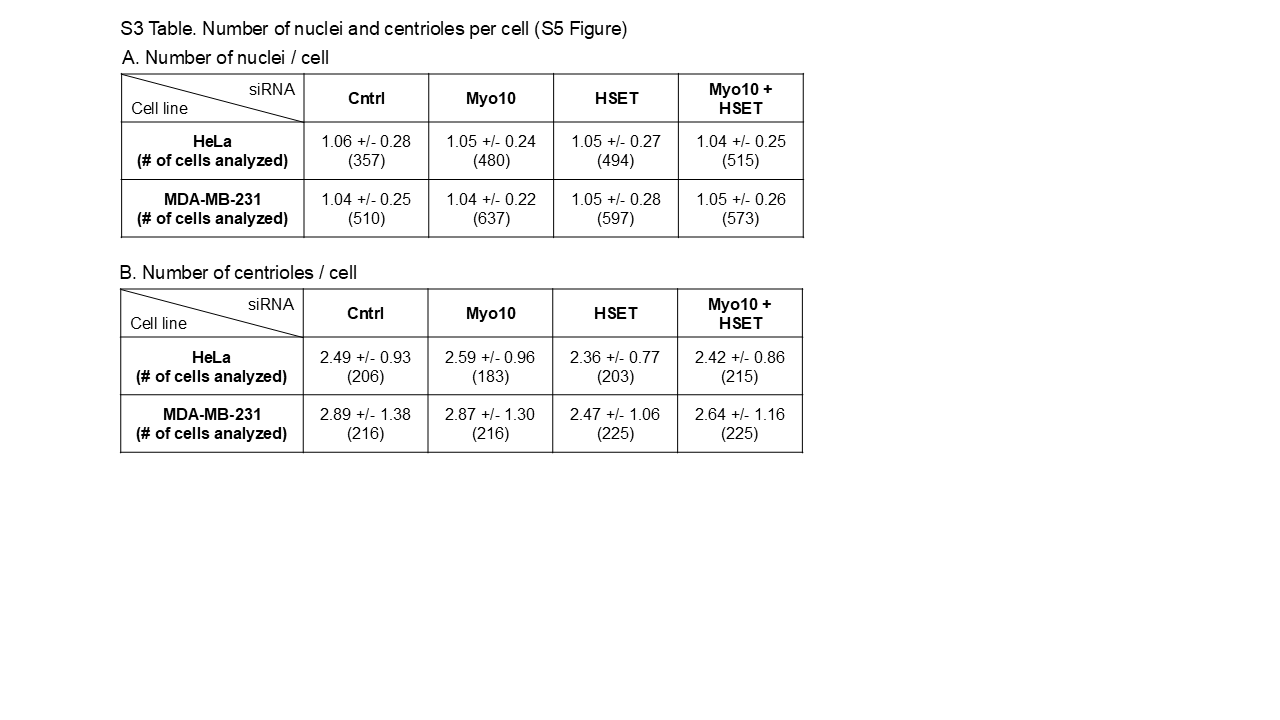

Supplement: S3 Table — This is the statistic outcomes corresponding to S5 Figure. A. Number of nuclei/ cell, B. Number of centrioles/ cell. (TIF) [file pone.0325016.s011.tif]

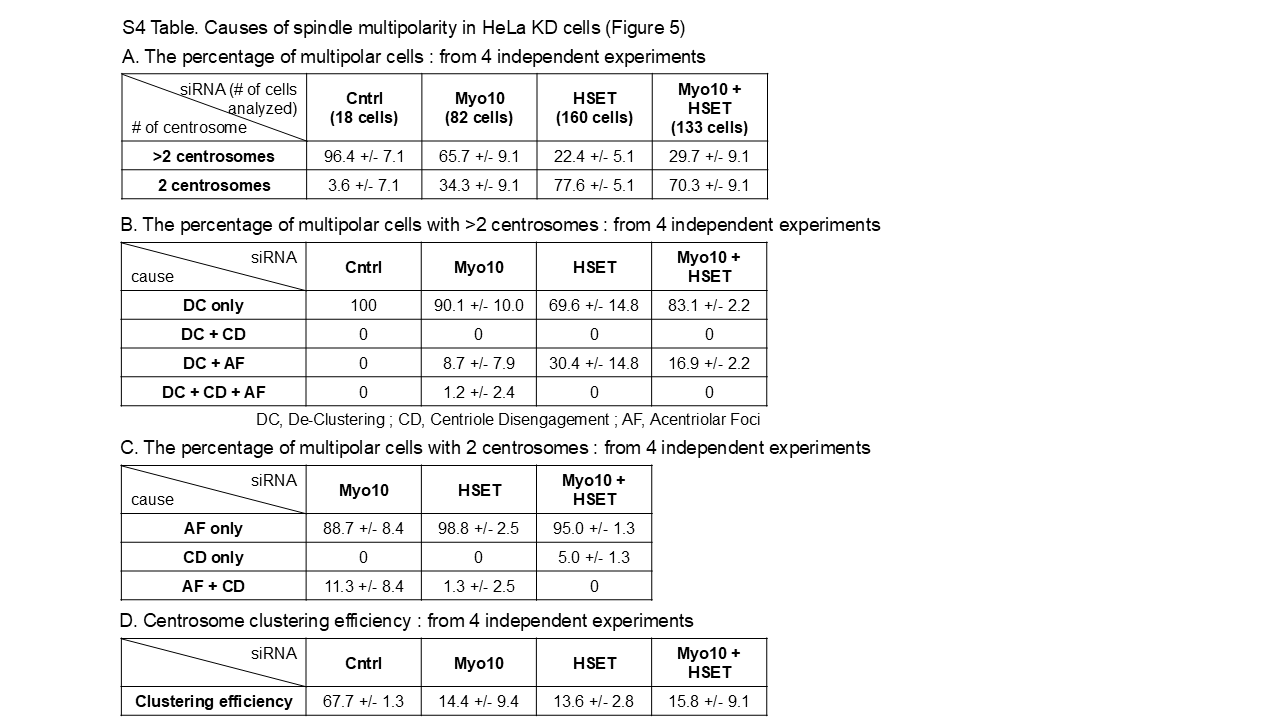

Supplement: S4 Table — This is the statistic outcomes corresponding to Figure 5. A. The percentage of multipolar cells: from 4 independent experiments, B. The percentage of multipolar cells with >2 centrosomes: from 4 independent experiments, C. The percentage of multipolar cells with 2 centrosomes: from 4 independent experiments, D. Centrosome clustering efficiency: from 4 independent experiments. (TIF) [file pone.0325016.s012.tif]

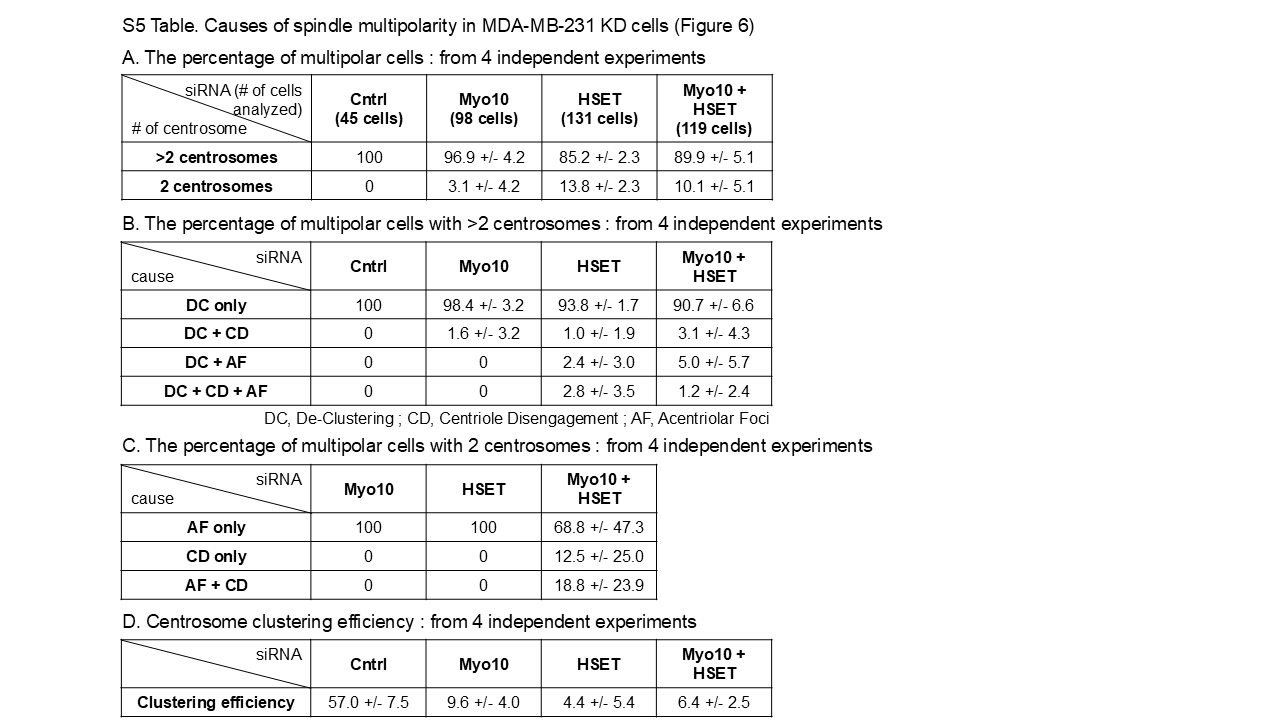

Supplement: S5 Table — This is the statistic outcomes corresponding to Figure 6. A. The percentage of multipolar cells: from 4 independent experiments, B. The percentage of multipolar cells with >2 centrosomes: from 4 independent experiments, C. The percentage of multipolar cells with 2 centrosomes: from 4 independent experiments, D. Centrosome clustering efficiency: from 4 independent experiments. (TIF) [file pone.0325016.s013.tif]

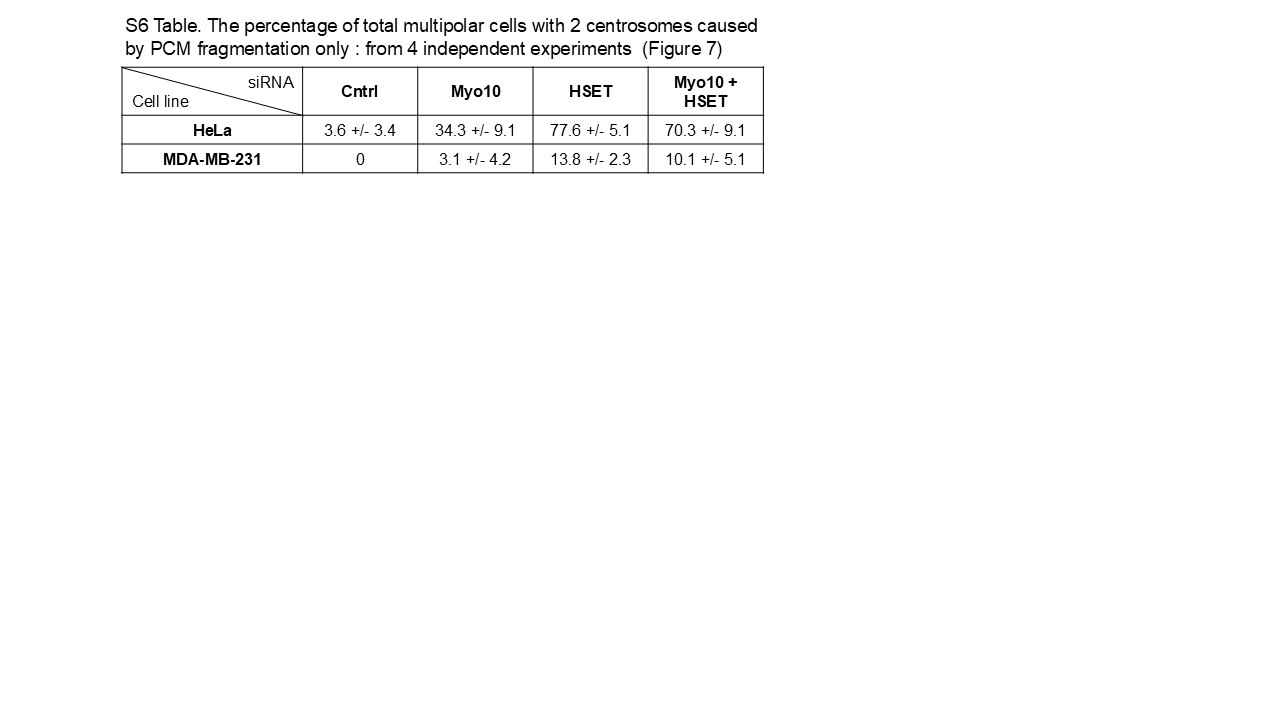

Supplement: S6 Table — This is the statistic outcomes corresponding to Figure 7. The results came from 4 independent experiments for both cell lines. (TIF) [file pone.0325016.s014.tif]

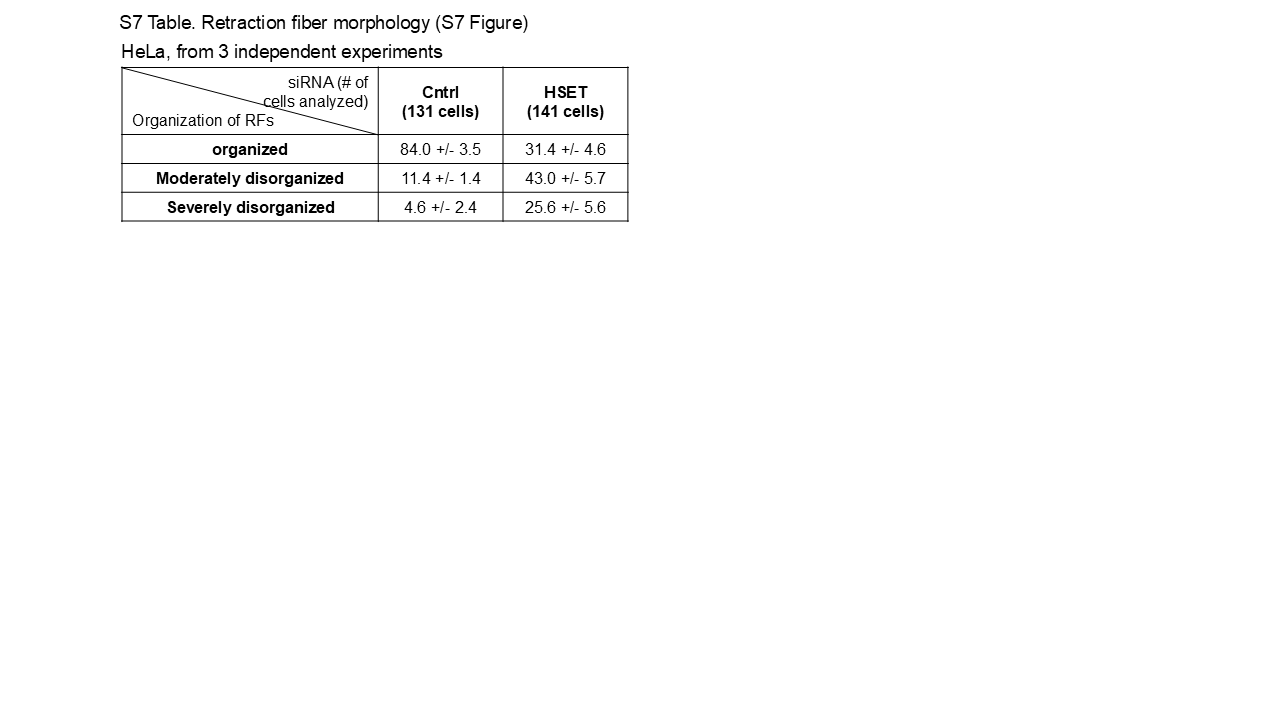

Supplement: S7 Table — This is the statistic outcomes corresponding to S7 Figure. The results came from 3 independent experiments in HeLa. (TIF) [file pone.0325016.s015.tif]

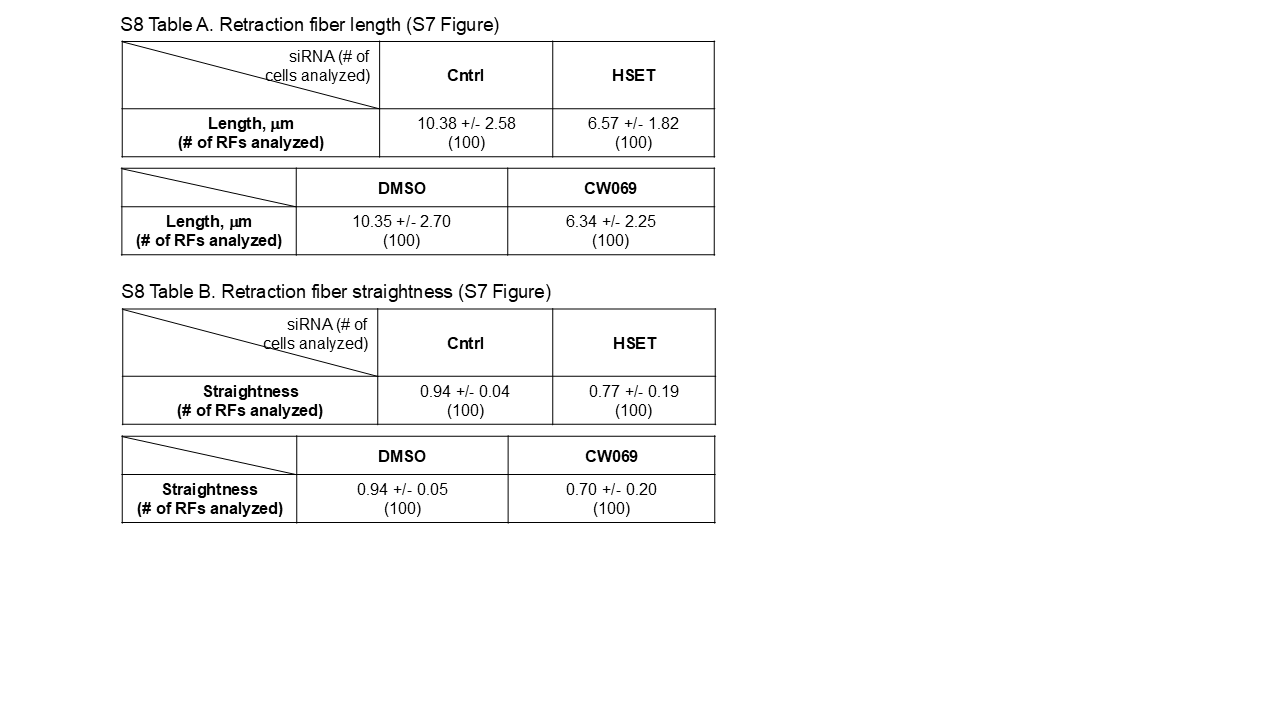

Supplement: S8 Table — A. Retraction fiber length, the statistic outcomes corresponding to S7 Figure D1 and D2, B. Retraction fiber straightness, the statistic outcomes corresponding to S7 Figure E1 and E2. (TIF) [file pone.0325016.s016.tif]

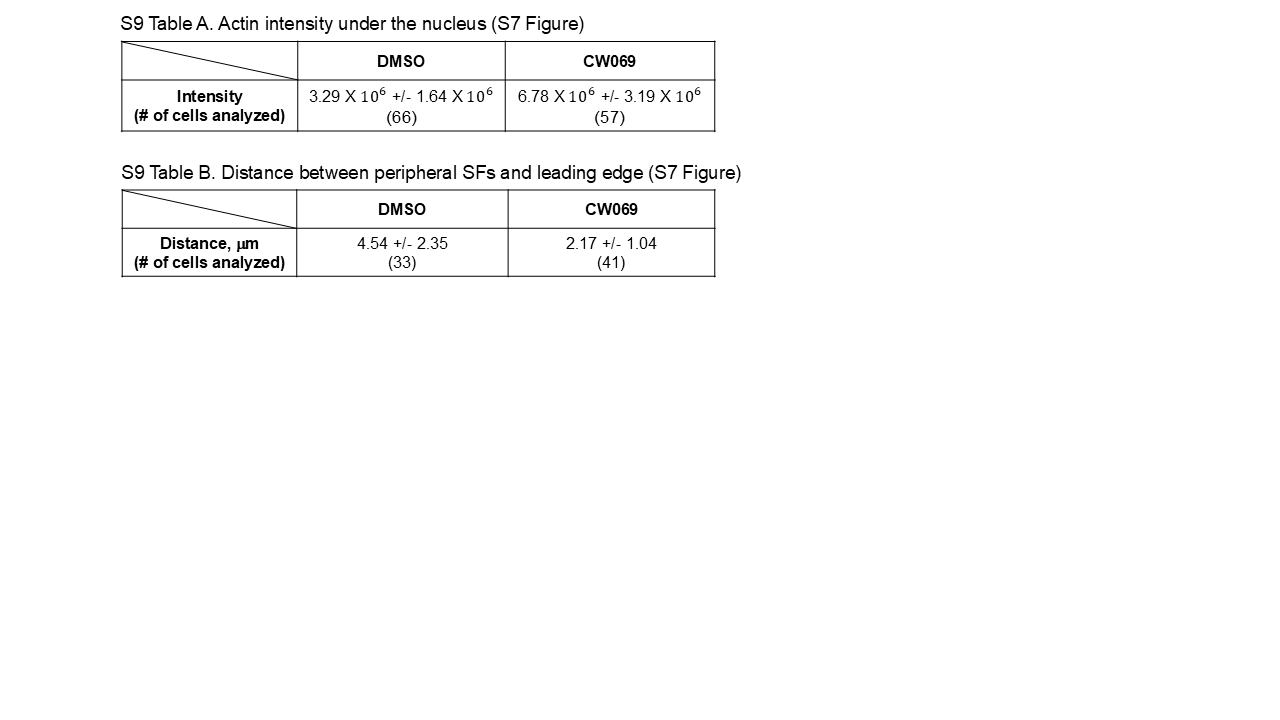

Supplement: S9 Table — A. Actin intensity under the nucleus, the statistic outcomes corresponding to S7 Figure H, B. Distance between peripheral SFs and leading edge, the statistic outcomes corresponding to S7 Figure I. (TIF) [file pone.0325016.s017.tif]

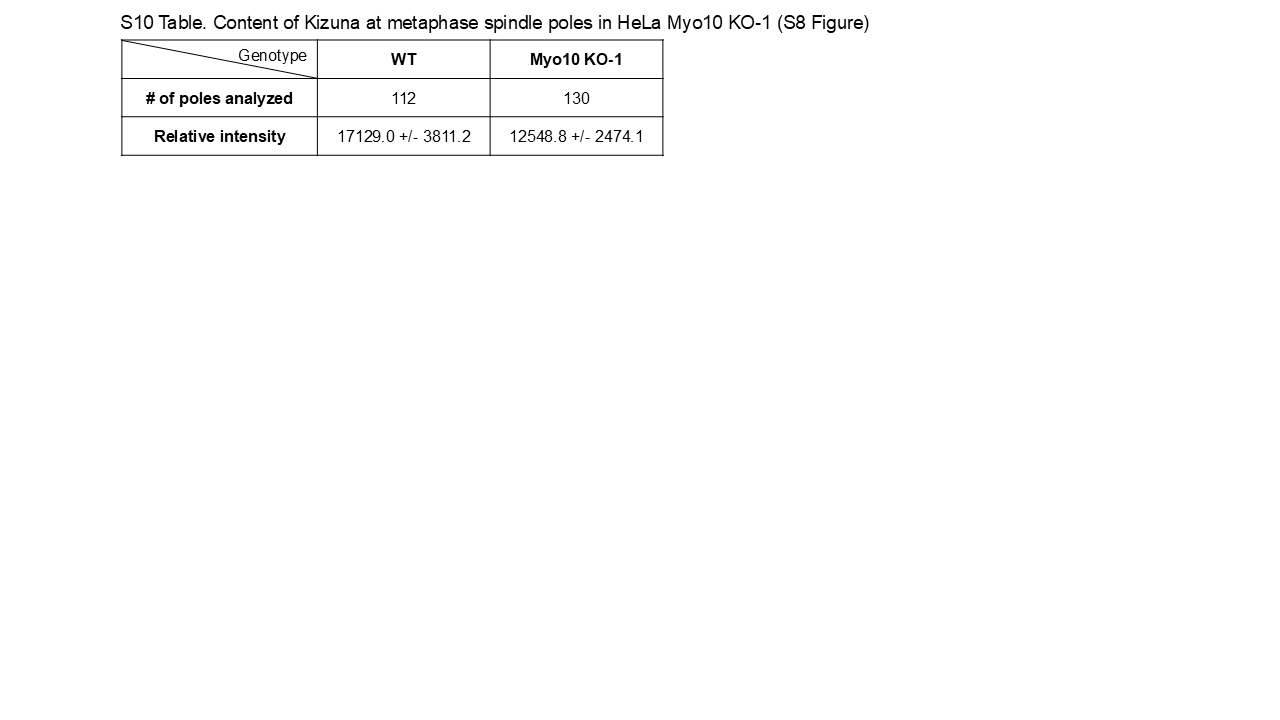

Supplement: S10 Table — This is the statistic outcomes corresponding to S8 Figure. (TIF) [file pone.0325016.s018.tif]

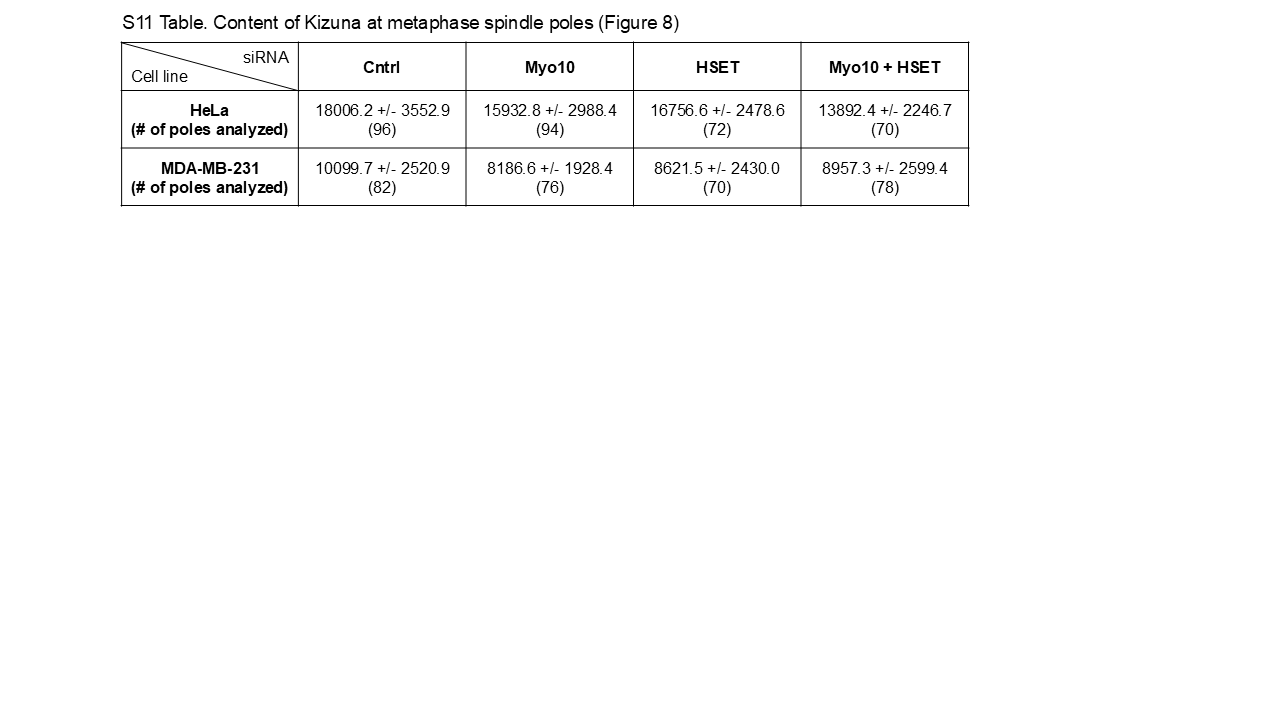

Supplement: S11 Table — This is the statistic outcomes corresponding to Figure 8. (TIF) [file pone.0325016.s019.tif]
